# 3D atomic structure determination with ultrashort-pulse MeV electron diffraction

**DOI:** 10.1107/S2052252526002782

**Published:** 2026-04-17

**Authors:** Vincent Hennicke, Max Hachmann, Paul Benjamin Klar, Patrick Y. A. Reinke, Tim Pakendorf, Jan Meyer, Hossein Delsim-Hashemi, Miriam Barthelmess, Sreevidya Thekku Veedu, Pontus Fischer, Ana C. Rodrigues, Arlinda Qelaj, Alexandra Tolstikova, Oleksandr Yefanov, Juna Wernsmann, Francois Lemery, Robin Schubert, Iñaki de Diego, Stuart Hayes, Sebastian Günther, Sven Falke, Erik Fröjd, Aldo Mozzanica, Lukas Palatinus, Kai Rossnagel, Bernd Schmitt, Henry N. Chapman, Wim Leemans, Klaus Flöttmann, Alke Meents

**Affiliations:** a Center for Free-Electron Laser Science CFEL Deutsches Elektronen-Synchrotron DESY Notkestr. 85 22607Hamburg Germany; b Deutsches Elektronen-Synchrotron DESY Notkestr. 85 22607Hamburg Germany; c Faculty of Geosciences and MAPEX Center for Materials and Processes University of Bremen Klagenfurter Str. 2 28359Bremen Germany; d European X-Ray Free-Electron Laser Facility GmbH, Holzkoppel 4, 22869Schenefeld, Germany; e Paul-Scherrer Institut Forschungsstrasse 111 5323Villigen Switzerland; f Institute of Physics of the Czech Academy of Sciences Na Slovance 2 182 21Prague 8 Czechia; g Institut für Experimentelle und Angewandte Physik Christian-Albrechts-Universität zu Kiel Olshausenstr. 40 24098Kiel Germany; h Ruprecht Haensel Laboratory Deutsches Elektronen-Synchrotron DESY Notkestr. 85 22607Hamburg Germany; i The Hamburg Centre for Ultrafast Imaging, Luruper Chaussee 149, 22761Hamburg, Germany; j Department of Physics University of Hamburg Luruper Chaussee 149 22761Hamburg Germany; Istituto Italiano di Tecnologia, Italy

**Keywords:** electron crystallography, high-energy electron diffraction, dynamical refinement, 2D materials, muscovite, tantalum disulfide

## Abstract

Understanding structure at the atomic scale is fundamental for understanding the functioning and the development of materials with improved properties. Compared with other probes providing atomic resolution, electrons offer the strongest interaction in combination with minimal radiation damage, which makes them an ideal tool for investigating very small and radiation-sensitive samples [Henderson (1995 ▸), *Q. Rev. Biophys.***28**, 171–193]. However, these benefits are often offset by the laborious preparation of nanometre-sized samples that are not visible using a light microscope, and the fact that experiments are largely restricted to ultra-high vacuum [Duyvesteyn *et al.* (2018 ▸), *Proc. Natl Acad. Sci. USA***115**, 9569–9573; Gruene *et al.* (2021 ▸), *Nat. Rev. Chem.***5**, 660–668]. Here, we report the successful implementation of MeV electron diffraction for *ab initio* 3D structure determination of the quasi-2D material muscovite and the quantum material 1*T*-TaS_2_ at atomic resolution. By employing ultrashort electron pulses from the REGAE (Relativistic electron gun for atomic exploration) accelerator, we obtained high-quality diffraction datasets suitable for structural refinements based on dynamical scattering theory, enabling precise localization of even hydrogen atoms. The increased penetration depth of MeV electrons significantly expands the applicable thickness range of samples, overcoming previous restrictions associated with traditional electron diffraction. These findings establish MeV electron diffraction as a viable approach for investigating a broad range of materials, including nano­structures and radiation-sensitive compounds, and open up new opportunities for *in situ* and time-resolved experiments [Chao *et al.* (2023 ▸), *Chem. Rev.***123**, 8347–8394; Filippetto *et al.* (2022 ▸), *Rev. Mod. Phys.***94**, 045004].

## Abstract 2

Using ultrashort-pulse MeV electron diffraction at the REGAE accelerator with increased penetration depth of the electrons significantly expands the applicable thickness range of samples, overcoming restrictions associated with traditional electron diffraction. We report the successful implementation of MeV electron diffraction for *ab initio* 3D structure determination of the quasi-2D material muscovite and the quantum material 1*T*-TaS_2_ at atomic resolution and open up new opportunities for *in situ* and time-resolved experiments.

## 1. Introduction

Understanding the three-dimensional structure of a material is crucial for unravelling its physical properties. Diffraction experiments using X-rays, neutrons or electrons are particularly well suited for determining structures with (sub)-atomic resolution.

Among these methods, electrons stand out as a superior probe owing to their significantly stronger elastic cross section and their ability to cause considerably less radiation damage to samples compared with X-rays (Henderson, 1995 ▸). Unlike X-rays, which primarily interact with electrons of the sample, electrons interact with the electrostatic potential. This interaction enhances the visibility of light elements, particularly hydrogen atoms (Palatinus *et al.*, 2017 ▸). Furthermore, highly brilliant electron beams are relatively easy to produce and can be precisely focused into submicron spot sizes using electromagnetic lenses. These features make electrons a uniquely economical and effective tool for structural investigations of submicron-sized samples, especially radiation-sensitive low-*Z* materials. As a result, electron diffraction has emerged as a rapidly growing research field (Gruene *et al.*, 2021 ▸). Today, three-dimensional electron diffraction (3D ED), also known as microcrystal electron diffraction (MicroED), is routinely used for structure determinations from nanocrystals. This technique is now being further advanced with the use of specialized devices designed specifically for electron crystallography (Nannenga *et al.*, 2014 ▸; Gemmi *et al.*, 2019 ▸; Ito *et al.*, 2021 ▸; Simoncic *et al.*, 2023 ▸).

The strong elastic interaction between electrons and matter, however, also leads to challenges in structure determination (Klar *et al.*, 2023 ▸). In particular, multiple scattering of electrons in the sample leads to non-kinematical diffracted intensities *I*_*hkl*_ so that the simple relationship with the squared structure-factor amplitudes, *I*_*hkl*_ ∝ |*F*_*hkl*_|^2^, does not hold. According to the well established dynamical theory of diffraction there is a non-linear dependence of *I*_*hkl*_ on experimental parameters such as the electron wavelength, crystal orientation, crystal shape and crystal thickness. Independent of that, electrons lose energy in the sample due to inelastic scattering events, which restricts the sample size for electron diffraction experiments. Depending on the density and chemical composition of the sample, the maximum tolerable thickness is typically limited to a few tens of nanometres in the case of inorganic (high-*Z*) samples and a few hundred nanometres for organic (low-*Z*) samples. These dimensions fall far below the resolution limit of optical microscopes, meaning that sample selection and preparation must be performed blindly, without the possibility of a straightforward light microscopic inspection before the experiment. This poses a significant limitation to the broader applicability of electron diffraction (ED) for structure determination (Duyvesteyn *et al.*, 2018 ▸; Bücker *et al.*, 2020 ▸).

Utilizing higher-energy electrons overcomes this limitation. Compared with 200 keV electrons, 3.48 MeV electrons have less than one-third of the elastic cross section, allowing for a more than threefold increase in permissible sample thickness (Henderson, 1995 ▸) while significantly reducing the impact of multiple scattering. As a result, organic and biological samples – previously limited to a maximum thickness of around 500 nm in conventional TEM devices – can now reach thicknesses of approximately 1.75 µm or even more in diffraction experiments using MeV electrons (Bozzola, 1992 ▸). This increase in tolerable sample thickness significantly broadens the applicability of ED to a much wider range of samples. Sample preparation steps, such as crystal growth, sample grinding and mounting samples on support structures, can now be performed under an optical microscope, similar to X-ray experiments.

**Figure 1:**
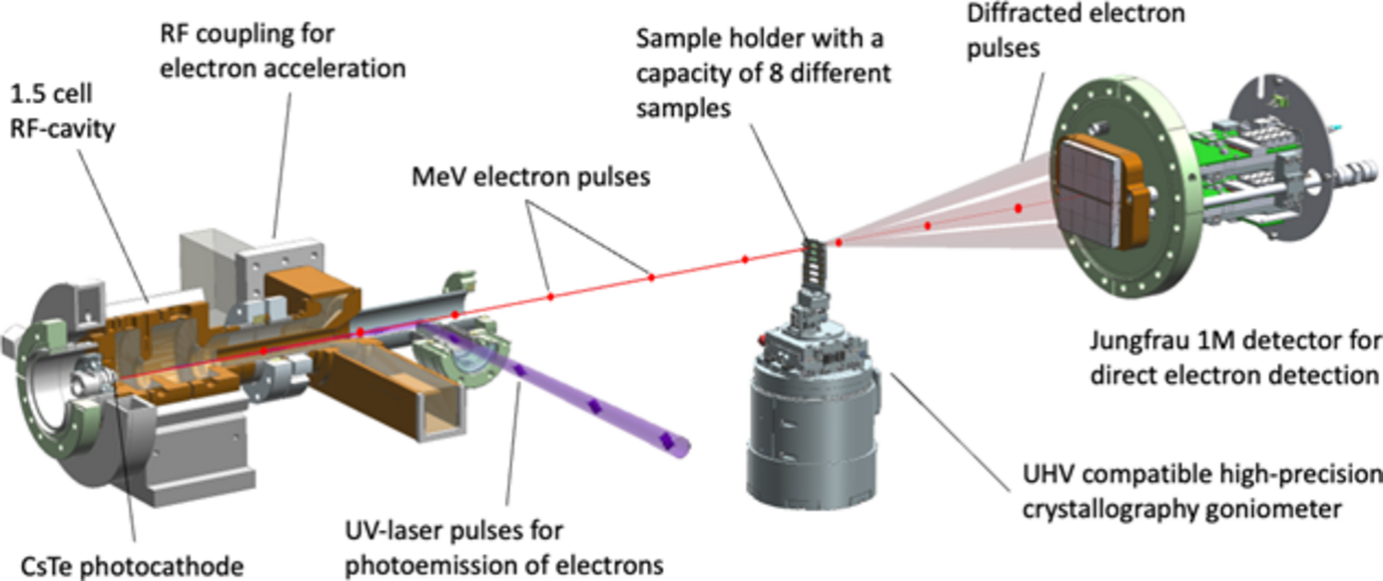
Concept of accelerator-based high-energy electron diffraction with MeV electrons. Electrons are emitted by illumination of the photocathode with short UV pulses, and are then immediately accelerated to energies of 2–5 MeV in a 1.5 cell RF cavity with field gradients of up to 110 MV m^−1^. The electron pulses (red dots) then interact with the sample, which is mounted on a single-axis goniometer for crystallographic data collection. While the sample is rotated in the electron beam, diffraction patterns are recorded on a Jungfrau 1M integrating pixel detector.

Furthermore, the longer focal lengths in high-energy diffraction devices provide a larger working space around the sample, enabling the use of a wide range of advanced and diverse sample environments (Fig. 1 ▸). This setup allows for *in situ* experiments over a broad temperature range – from milli-Kelvin to several thousand Kelvin – as well as the use of different gas atmospheres, which is particularly advantageous for catalysis research. The sample holder and space for sample environments of a typical TEM, in contrast, are limited to dimensions in the millimetre range and can reach a few centimetres for dedicated electron diffraction devices operating at energies of up to 160 keV.

Moreover, high-energy MeV electrons are far less affected by electric and magnetic fields, enabling experiments that are difficult or impractical with lower-energy electrons; thereby further broadening their potential applications.

However, achieving electron energies of several MeV with the beam stability and coherence length necessary for high-quality structure determination in electron crystallography has been experimentally challenging. For instance, in a transmission electron microscope, accelerating electrons to 1 MeV in static DC fields requires a complex 36-stage accelerator tube (Kawasaki *et al.*, 2000 ▸).

A more promising approach is the use of well established radio frequency (RF)-based accelerator technology. Unlike conventional acceleration in a static field, RF-based accelerators deliver temporally short electron pulses with lengths varying between a few hundred picoseconds down to a few femtoseconds (Weathersby *et al.*, 2015 ▸; Zeitler *et al.*, 2015 ▸). Such ultrashort probe pulses in combination with a short wavelength enable femtosecond time-resolved experiments with atomic resolution, which so far are predominantly conducted at much larger X-ray free-electron laser (XFEL) facilities.

Ultrashort, high-energy electron pulses have already proven highly effective in time-resolved ultrafast electron diffraction (UED) experiments. Beyond their application to solids and crystalline samples, UED techniques have also been successfully employed to study liquid and gaseous samples (Kogar *et al.*, 2020 ▸; Nunes *et al.*, 2020 ▸; Lin *et al.*, 2021 ▸). However, the application of UED for 3D structure determination at atomic resolution has been postulated to be unfeasible owing to limitations in adequate sampling of reciprocal space (Ishikawa *et al.*, 2015 ▸). In fact, no diffraction experiments with ultrashort electron pulses that provided *ab initio* 3D structural information have yet been reported.

Here we report the first successful *ab initio* 3D structure determination at atomic resolution of the quasi-2D layered materials muscovite and the 1*T* phase of tantalum di­sulfide using the ‘Relativistic electron gun for atomic exploration – (REGAE)’ facility at DESY. Muscovite is a layer silicate and has long been used as a very thin electrical insulator (Liang *et al.*, 1998 ▸; Gatta *et al.*, 2011 ▸). 1*T*-TaS_2_ belongs to the class of transition metal dichalcogenides (TMDCs) – a technologically highly relevant class of 2D quantum materials, which exhibit structurally very exciting properties such as charge-density waves (CDWs) and undergo several phase transitions (Spijkerman *et al.*, 1997 ▸; Haupt *et al.*, 2016 ▸; Manzeli *et al.*, 2017 ▸).

## 2. Experimental

Additional detailed information on instrumentation, materials and methods is provided in the supporting information.

### 2.1. REGAE accelerator facility

Diffraction experiments were performed at the ‘Relativistic gun for atomic exploration – REGAE’ facility at DESY in Hamburg, Germany (Manz *et al.*, 2015 ▸). REGAE is a linear accelerator facility explicitly designed and built for time-resolved diffraction experiments with MeV electrons (Fig. 1 ▸, Fig. S1). At REGAE, electrons are generated by photoemission inside an S-band (3 GHz) RF gun and rapidly accelerated to an energy of 3 to 5 MeV using field gradients of up to 110 MV m^−1^ on the photocathode.

A cathode exchange system at REGAE allows selection between different photocathodes without breaking the UHV. For the current work, a CsTe cathode providing high quantum efficiency was used without any specific reasons. REGAE is capable of generating electron pulses at a repetition rate of 50 Hz and with typical bunch charges of up to 100 fC with a transverse emittance as low as 10 nm, which yields a lateral coherence length of about 2–10 nm at typical transverse r.m.s. beam sizes of 50–250 µm on the sample (Hachmann & Flöttmann, 2016 ▸).

### 2.2. UHV compatible diffraction setup

For diffraction experiments REGAE is equipped with a dedicated UHV-compatible diffraction setup consisting of an in-line sample viewing microscope and a high-precision goniometer for crystallographic data collection (Figs. 1 ▸, S2, S3, S4, S5). The design was inspired by existing in-air X-ray diffraction setups commonly used at synchrotron sources (Burkhardt *et al.*, 2016 ▸).

For sample visualization and beamline alignment an in-line sample viewing microscope can be inserted into the electron beam path, allowing the user to permanently visualize the sample with optical light during the diffraction experiment. The in-line microscope allows the sample to be viewed with visible light collinear to the electron beam. For this, the electron beam passes through a 1.0 mm drill hole along the optical axis of the microscope objective.

The e^−^-Roadrunner goniometer is a compact and UHV-compatible single-axis crystallographic goniometer with a high-precision vertical rotation axis. The goniometer axis itself consists of a directly-servomotor-driven and UHV-compatible rotation axis based on ceramic bearings providing 360° rotation capability with an angular resolution of 0.001° in combination with a sphere of confusion smaller than 1 µm. A motorized centring stage mounted on top of the rotation axis provides travel ranges of ±6 mm in the *x*, *y* and *z* directions, and allows precise positioning of the sample in the centre of the rotation axis.

### 2.3. Jungfrau 1M detector for direct electron detection

Diffraction data are recorded with an in-vacuum version of a Jungfrau 1M detector (Fig. S7). The detector was originally developed for experiments with high-intensity X-ray pulses at XFELs (Mozzanica *et al.*, 2018 ▸; Leonarski *et al.*, 2018 ▸). In contrast to indirect, scintillator-based detectors, the Jungfrau detector directly records the electrical signal generated by the inelastic interactions of the electrons within the silicon sensor material, a signal that is proportional to the energy deposited in the sensor and which, given the high peak current, allows a higher signal-to-noise ratio. In addition, a gating function records data only during a very short period centred around the arrival time of the electron pulse. All other signals, for example dark current from the accelerator, arriving before and after the gating period are not recorded and hence do not contribute to the background signal in the data.

The Jungfrau detector is composed of two 500 kpixel modules and can be operated at frame rates of up to 2 kHz. By using an automatic in-pixel gain selection with three different feedback capacitors, it provides a sufficient dynamic range for detecting strong Bragg reflections of up to 1200 electrons at 3.48 MeV per pixel per pulse.

### 2.4. Muscovite and TaS_2_ samples

Muscovite, with chemical composition KAl_3_Si_3_O_10_(OH)_2_, crystallizes in space group *C*2/*c* with unit-cell parameters *a* = 5.21, *b* = 9.04, *c* = 20.03 Å and β = 95.8° (Gatta *et al.*, 2011 ▸). Sheets of corner-sharing tetrahedra and edge-sharing octahedra are oriented perpendicular to the crystallographic *b* axis, resulting in extremely good cleavability between the layers. Muscovite samples were prepared by using the technique of exfoliation (Fig. S8).

The 1*T*-phase of TaS_2_ used for our experiments is described in the space group 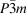 with unit-cell parameters *a* = 3.365 Å and *c* = 5.883 Å (Spijkerman *et al.*, 1997 ▸). A charge-density wave is formed by periodic, static displacements of Ta and S, giving rise to additional Bragg satellite reflections in the diffraction patterns. The incommensurately modulated structure was previously described with the superspace group *X*3(α, β, 0)0(−α − β, α, 0)0 (Spijkerman *et al.*, 1997 ▸). 1*T*-TaS_2_ samples were prepared by microtome cutting perpendicular to the naturally occurring layers oriented parallel to the crystallographic *ab* plane (Fig. S9) (Haupt *et al.*, 2016 ▸).

### 2.5. MeV electron diffraction data collection

Electron diffraction data for muscovite and 1*T*-TaS_2_ were recorded at REGAE at an energy of 3.48 MeV and a pulse duration of 600 fs at room temperature (293 K). No active beam stabilization based on beam diagnostics was used for measurements. To still achieve a high beam stability, only environmental parameters such as humidity and temperature were well controlled and the RF system was actively stabilized.

**Figure 2:**
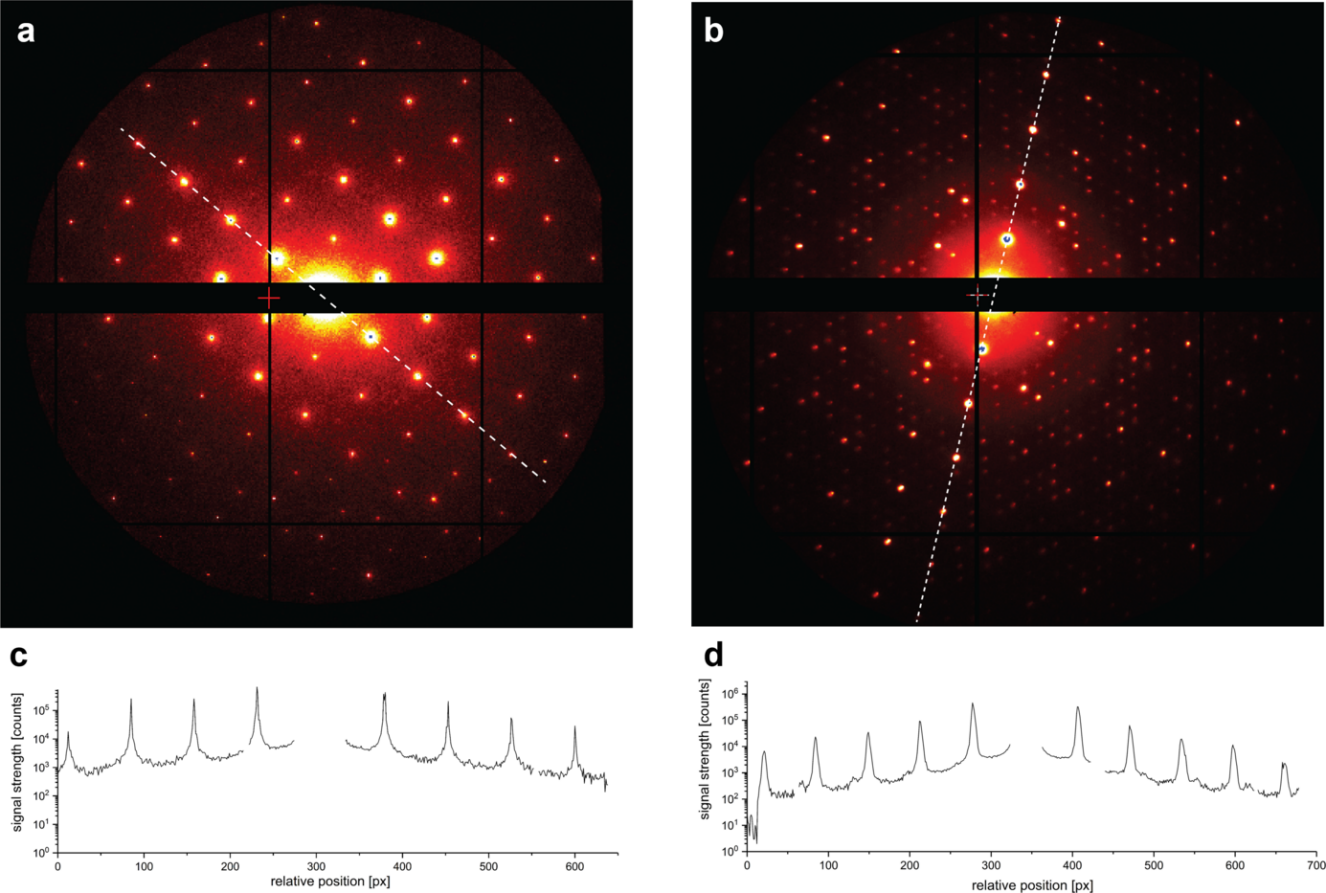
MeV electron diffraction patterns. Sum of 12 single-shot diffraction still images recorded with 3.48 MeV high-energy electron pulses at the REGAE facility from muscovite (*a*) and 1*T*-TaS_2_ (*b*) at a pulse rate of 12.5 Hz. The corresponding intensity profiles along the dashed lines indicated in the diffraction patterns highlight the achievable signal-to noise-ratio resulting from the high coherence of the electron beam and the low background scattering signal [(*c*) and (*d*)].

For data collection, the samples were rotated from −60° to +60° in discrete steps of 0.01° for muscovite and from −65° to +65° in discrete steps of 0.005° for 1*T*-TaS_2_, resulting in a pseudo-continuous rotation (Table S1). For each angular position, diffraction still images from 12 electron pulses were recorded. In total 153 600 diffraction images were recorded from muscovite and 312 000 diffraction images from TaS_2_, corresponding to total data collection times of about 3.5 h and 7.5 h, respectively. The diffraction images clearly show sharp Bragg reflections and very low noise levels between the reflections, highlighting the excellent achievable signal-to-noise ratio (Fig. 2 ▸). For 1*T*-TaS_2_, weaker reflections around the Bragg peaks originating from the CDW can be clearly observed. The well resolved spatial separation of the reflections is a clear indication of the high transverse and longitudinal coherence lengths of the electron bunches from REGAE.

**Figure 3:**
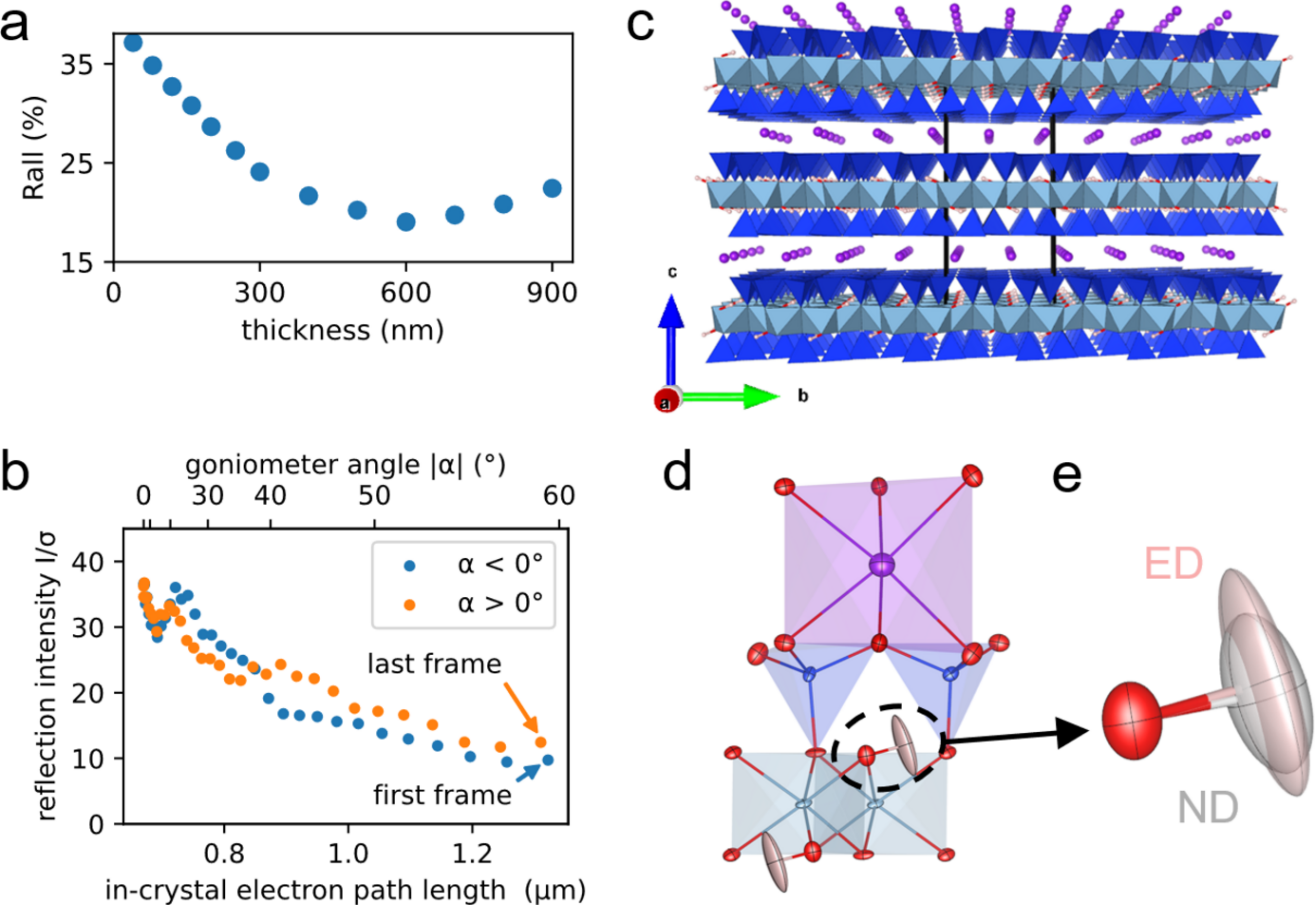
Results from dynamical structure refinement of the muscovite high-energy electron diffraction data. (*a*) Dependence of the *R*_all_ value from dynamical structure refinements as function of the adopted sample thickness. (*b*) Decay of the mean *I*/σ(*I*) ratio in the diffraction data as function of the increasing effective sample thickness caused by sample rotation and the corresponding goniometer angle. (*c*) View of the muscovite layer structure along the crystallographic *a* axis. (*d*) Detailed view of the muscovite structure with anisotropic thermal displacement parameters indicated as ellipsoids (potassium = violet, aluminium = blue, oxygen = red, hydrogen = pink). (*e*) Overlay of the OH group with anisotropic thermal displacement parameters shown as ellipsoids obtained with electron diffraction (ED, this work) and a reference model obtained with neutron diffraction (ND) (Gatta *et al.*, 2011 ▸).

With a bunch charge of 60 fC and a beam diameter of 500 µm, an integrated fluence of 2.8 × 10^−3^ e^−^ Å^−2^ was delivered to the central part of the muscovite sample. The integrated fluence for the 1*T*-TaS_2_ sample using pulse charges of less than 15 fC and a beam diameter of 50 µm was less than 0.14 e^−^ Å^−2^. During data collection, we could not detect any decay in the diffraction signals and quality, indicating an absence of radiation-damage effects for the radiation doses applied here [Fig. 3 ▸(*b*)].

### 2.6. Data reduction and structure solution

Data reduction was performed with the software *PETS2* (Palatinus *et al.*, 2019 ▸). From *PETS2* data processing a beam stability of about ±1 detector pixel (0.075 mm) in the *x* direction (horizontal) and ±2 pixels in the *y* direction (vertical) at the position of the Jungfrau detector was derived over the entire measurement time for each dataset. The quality of the diffraction datasets allowed a straightforward structure solution of the muscovite structure and the average 1*T*-TaS_2_ structure using well established software (Palatinus & Chapuis, 2007 ▸; Sheldrick, 2008 ▸; Burla *et al.*, 2015 ▸). The (3+2)*d* superspace structure of 1*T*-TaS_2_ could be solved with *SUPERFLIP* (Palatinus & Chapuis, 2007 ▸). Initial kinematical refinements, neglecting multiple scattering events, were performed with *Jana2020* (Petříček *et al.*, 2023 ▸). They yielded *R*_all_ values of 17.3% for muscovite and 25.5% for the modulated room-temperature structure of 1*T*-TaS_2_. To obtain a better agreement between the measured and calculated structure amplitudes and hence a better model of the structures, *Jana2020* was also used to carry out dynamical structure refinements (Klar *et al.*, 2023 ▸; Palatinus, Petříček & Corrêa, 2015 ▸; Palatinus, Corrêa *et al.*, 2015 ▸).

## 3. Results

### 3.1. Muscovite

Dynamical refinement against the muscovite dataset resulted in an improved merged *R*_all_ value of 9.4% (Table S3). The refinement appeared highly sensitive to the assumed crystal thickness and crystal mosaicity. The *R*_all_ values from the refinements show a distinct minimum for a thickness around 650 nm. When freely refined, a crystal thickness of 634 nm is obtained [Fig. 3 ▸(*a*)], which agrees well with the measured thickness of 670 nm. During data collection, the effective sample thickness, *i.e.* the length of the electron path through the crystal, increases up to 1.4 µm at a rotation angle of 60°. But even at this large thickness, excellent data quality with a reasonable *I*/σ(*I*) ≃ 10 could be obtained [Fig. 3 ▸(*b*)].

In the resulting structural model, all details are well resolved and meaningful anisotropic thermal displacement parameters are obtained [Figs. 3 ▸(*c*), 3 ▸(*d*); data in CIF format are available in the supporting information]. A comparison of atom-site coordinates with a reference structure of slightly different chemical composition (Gatta *et al.*, 2011 ▸) using the tool *COMPSTRU* yielded an average deviation of 0.032 Å with a maximum deviation of 0.083 Å for the non-hydrogen sites (de la Flor *et al.*, 2016 ▸). The high quality of the diffraction data in combination with the high resolution in reciprocal space accessible with our method even allows the identification and free refinement of the hydrogen-atom position with meaningful anisotropic thermal displacement parameters. The parameters obtained for the hydrogen atom and the resulting O—H bond length of 0.95 (4) Å and the ADPs agree very well with neutron reference data, stating a length of 0.939 (5) Å [Fig. 3 ▸(*e*), Table S4] (Gatta *et al.*, 2011 ▸). A comparative X-ray structure determination conducted from exactly the same sample as used for our MeV ED experiments yielded an O—H bond length of 0.81 (6) Å and did not allow a free refinement of the ADPs within the independent-atom model.

### 3.2. 1*T*-TaS_2_

**Figure 4:**
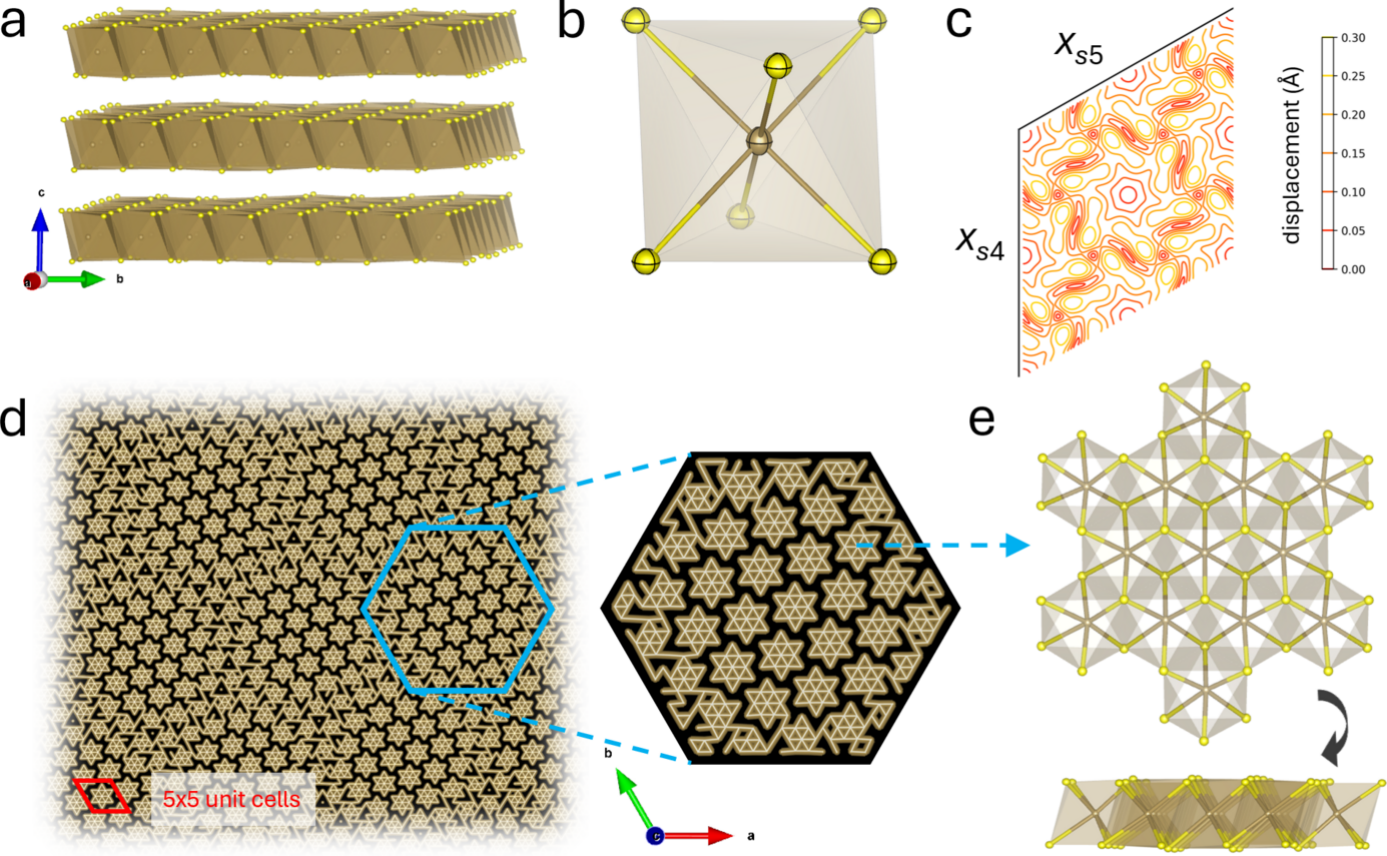
TaS_2_ structure as determined by MeV electron diffraction at REGAE. (*a*) TaS_2_ layer structure viewed along the crystallographic *a* axis. (*b*) Octahedral coordination of the Ta atom by six sulfur atoms with thermal displacement parameters represented as ellipsoids. (*c*) Refined modulation displacement amplitudes of the Ta atom as a function of *x*_*s*4_ and *x*_*s*5_ analogous to the analysis by Spijkerman *et al.* (1997 ▸). (*d*) Real-space representation of the modulated structure of ∼75 × 75 crystallographic unit cells showing the arrangement of star-shaped Ta clusters, each consisting of 13 tantalum atoms. Ta atoms separated by less than 3.4 Å are connected by lines. Sulfur atoms are omitted. (*e*) View of a single star-shaped 13-atom tantalum cluster together with the coordinating sulfur atoms viewed along **c** (top) and rotated by 90° about **a** (bottom).

Dynamical refinement of the 1*T*-TaS_2_ dataset resulted in an improved merged *R*_all_ value of 4.3% for the main reflections and 12.0% for all reflections including the satellite reflections of 1*T*-TaS_2_ (Table S5). Similar to muscovite, all structural details are well resolved, and meaningful thermal displacement parameters are obtained [Fig. 4 ▸(*a*, *b*); data in CIF format are available in the supporting information]. The weak satellite reflections originating from the charge-density wave were about 1–2 orders of magnitude lower in counts than the main structural reflections. Including these in the dynamical structure refinement enabled us to refine the incommensurate structure of 1*T*-TaS_2_ [Fig. 4 ▸(*c*)]. In agreement with a previous study conducted with X-rays from a 300× thicker single crystal, an arrangement of star-like tantalum clusters within the layers is observed, each consisting of 13 tantalum atoms [Figs. 4 ▸(*d*), 4 ▸(*e*)] (Spijkerman *et al.*, 1997 ▸).

## 4. Conclusions and outlook

We have successfully applied accelerator-based high-energy electron diffraction to the field of 3D crystallographic structure determination, which has traditionally been dominated by X-rays. By integrating cutting-edge accelerator science with novel diffraction instrumentation, a direct electron-detection-capable detector, optimization of dark current and reduction of background scattering – paired with the latest software advances for dynamical diffraction data analysis – we were able to solve and refine the structure of the quasi-2D layered materials muscovite and 1*T*-TaS_2_*ab initio* with dynamical refinements at very high quality, as clearly indicated by very low merged *R*_all_ values of 9.4% for muscovite and 4.3% for the unmodulated structure of TaS_2_.

Electron energies exceeding 1 MeV – specifically 3.48 MeV in our case – strike an optimal balance between penetration depth and scattering contrast, making them ideal for structural studies of micro- and nanocrystals. For muscovite, the reduced elastic cross sections of high-energy electrons enabled us to acquire high-quality diffraction data from a 670 nm thick sample, an achievement unattainable with current lab-based electron diffraction devices or electron microscopes. For proteins, which have a density approximately 2.5 times lower than muscovite, theoretical predictions and microscopy experiments on biological specimens (Cosslett, 1969 ▸) suggest that it should be possible to analyse crystals up to 2 µm in size. These would be easily visible under an optical microscope, significantly simplifying and streamlining sample preparation processes.

At the same time, the elastic cross section at 3.48 MeV remains sufficiently high to enable high-quality structure determination from samples as thin as 30 nm. This capability allowed us to precisely resolve the incommensurate structure of 1*T*-TaS_2_ using satellite reflections that are, on average, 1–2 orders of magnitude weaker than the main reflections. Given that the TaS_2_ measurements utilized only a small fraction of the available bunch charge, it should be entirely feasible to perform diffraction experiments on just a few or even single layers of quantum materials at REGAE.

A current limitation of the method is the transversely large electron beam size caused by space charge effects, necessitating the use of laterally large yet relatively thin pancake-like samples. To address this, we are implementing a bunch-train mode at REGAE (Mahnke *et al.*, 2024 ▸), in which the bunch charge of up to 100 fC is evenly distributed across 4500 microbunches with a total duration of 1.5 µs. This approach enables the electron beam to be focused down to a few micrometres, allowing the investigation of isometric microcrystals of comparable size while preserving its excellent coherence properties.

For radiation-sensitive samples such as proteins, the larger penetration depth of MeV electrons, combined with their large scattering cross section and expected reduced radiation-damage effects, should allow routine collection of a complete dataset suitable for structure determination from a single micro- or nanocrystal, which is in most cases not possible with X-rays (Holton, 2009 ▸). This should make high-energy electrons the ideal probe for structure determination of such small crystals, for example in pharmaceutical compound screening experiments in the framework of structure-based drug discovery (Günther *et al.*, 2021 ▸).

In addition, the more realistic hydrogen-atom positions obtained in electron diffraction experiments, as shown here for muscovite, should lead to a better understanding of enzyme and other catalytic reactions, where hydrogen bonding and transfer reactions play a very important role (Herschlag & Pinney, 2018 ▸).

One parameter not utilized in the present work is the high temporal resolution, down to the single-digit femtosecond range, achievable at linear accelerators such as REGAE. Currently, experiments that combine sub-atomic spatial resolution with femtosecond temporal resolution are predominantly conducted at XFEL sources. However, with significantly reduced radiation-damage effects, such UED experiments at accelerator facilities could enable similar investigations without requiring continuous sample replenishment. This approach would, for instance, allow the study of coherent phonons and ultrafast phase transitions in single layers of 2D quantum materials like 1*T*-TaS_2_ and other transition-metal dichalcogenides with exceptional sensitivity (Filippetto *et al.*, 2022 ▸).

With significantly lower investment and operating costs – particularly in terms of electricity consumption – accelerator-based electron diffraction experiments using MeV electrons present an economically and ecologically promising approach for structural and dynamical investigations of micro- and nanometre-sized materials. These methods serve as a valuable complement to existing microfocus synchrotron beamlines and XFELs.

## 5. Related literature

The following references are cited in the supporting information: Bourhis *et al.*, 2015 ▸; Brázda *et al.*, 2022 ▸; Catti *et al.*, 1994 ▸; Fröjdh *et al.*, 2022 ▸; Kabsch, 2010 ▸; Khouchen *et al.*, 2023 ▸; Novoselov *et al.*, 2012 ▸; Redford *et al.*, 2018 ▸; Sheldrick, 2015 ▸; Stokes *et al.*, 2011 ▸; van Smaalen *et al.*, 2013 ▸.

## Supplementary Material

Crystal structure: contains datablock(s) muscoviteElectron, muscoviteXray, TaS2. DOI: 10.1107/S2052252526002782/of5008sup1.cif

Structure factors: contains datablock(s) global, muscoviteElectron. DOI: 10.1107/S2052252526002782/of5008muscoviteElectronsup2.hkl

Structure factors: contains datablock(s) muscoviteXray. DOI: 10.1107/S2052252526002782/of5008muscoviteXraysup3.hkl

Structure factors: contains datablock(s) global, TaS2. DOI: 10.1107/S2052252526002782/of5008TaS2sup4.hkl

Supporting tables and figures, and full movie captions. DOI: 10.1107/S2052252526002782/of5008sup5.pdf

Movie S1. Animated de Wolff section (xs1-xs4) of the Ta site. DOI: 10.1107/S2052252526002782/of5008sup6.mp4

Movie S2. Animated de Wolff section (xs2-xs4) of the Ta site. DOI: 10.1107/S2052252526002782/of5008sup7.mp4

Movie S3. Animated de Wolff section (xs3-xs4) of the Ta site. DOI: 10.1107/S2052252526002782/of5008sup8.mp4

Movie S4. Animated de Wolff section (xs1-xs4) of the S site. DOI: 10.1107/S2052252526002782/of5008sup9.mp4

Movie S4. Animated de Wolff section (xs2-xs4) of the S site. DOI: 10.1107/S2052252526002782/of5008sup10.mp4

Movie S6. Animated de Wolff section (xs3-xs4) of the S site. DOI: 10.1107/S2052252526002782/of5008sup11.mp4

IAyACLYazaI

CCDC references: 2538230, 2545330
