# Supplementary material for: 3D atomic structure determination with ultrashort-pulse MeV electron diffraction
Source: IUCrJ. 2026 Apr 17;13(Pt 3):282–90. doi: 10.1107/S2052252526002782 (PMC13134489; doi:10.1107/S2052252526002782)
Supplement: Supplementary file 5 [file m-13-00282-sup5.pdf]

# IUCrJ

Volume 13 (2026)


**Supporting information for article:**

## 3D atomic structure determination with ultrashort-pulse MeV electron diffraction

Vincent Hennicke, Max Hachmann, Paul Benjamin Klar, Patrick Y. A. Reinke, Tim Pakendorf, Jan Meyer, Hossein Delsim-Hashemi, Miriam Barthelmess, Sreevidya Thekku Veedu, Pontus Fischer, Ana C. Rodrigues, Arlinda Qelaj, Alexandra Tolstikova, Oleksandr Yefanov, Juna Wernsmann, Francois Lemery, Robin Schubert, Iñaki de Diego, Stuart Hayes, Sebastian Günther, Sven Falke, Erik Fröjd, Aldo Mozzanica, Lukas Palatinus, Kai Rossnagel, Bernd Schmitt, Henry N. Chapman, Wim Leemans, Klaus Flöttmann and Alke Meents

## S1. REGAE accelerator

REGAE provides short pulses of electrons over an energy range of 3-5 MeV with bunch charges of up to 100 fC and pulse durations down to 20 fs (rms). Electrons are emitted from a photocathode upon irradiation with a short UV laser pulse and directly accelerated in a 3 GHz RF-gun (S-band) with field gradients of up to 110 MV/m on the cathode. These electron pulses with a typical duration of several hundreds of femtoseconds can then be further longitudinally compressed using a buncher cavity leading to pulse duration down to 20 fs (rms). A technical overview drawing of the REGAE facility is provided in Figure S1.

Several collimation systems in the electron beam path allow to additionally shape the electron beam and scrape off halo electrons and dark-current electrons. The lengths of the REGAE accelerator part as measured from the photocathode to the sample position is 5.5 m (Fig. S2). With an additional propagation length of 5.43 m between the sample position and the detector the overall length of the REGAE experiment adds up to 10.9 m.

## S2. Laser system

A Titan-sapphire laser (model: Legend, vendor: Coherent) system is utilized for both electron generation in the photocathode and optionally also for time-resolved experiments for sample excitation in the UV/VIS range. For synchronization to the RF-system the oscillator cavity of the laser is synchronized to the master frequency of REGAE by a direct conversion system with a temporal stability of 11 fs (rms). After passing through the amplifier the 800 nm laser light is split into two beams. A small fraction (< 10%) of the beam is frequency-tripled and then directly guided to the photocathode for electron generation (probe pulse). The larger fraction can be utilized for sample excitation (pump pulse). The path lengths of the two laser beams defines the arrival time at the corresponding target (photocathode and sample). Typically, a shorter beam path is required for sample excitation, so that the sample is excited before arrival of the probing electron pulse. Variation of the path length of the pump laser beam allows to adjust the time delay between the two beams.

## S3. Electron gun

REGAE is equipped with a load lock system allowing for fast exchange of the photocathode und ultra-high vacuum (UHV). For the present experiment Mo was used as photocathode material. The RF gun is a 1.5 cell normal conducting copper cavity operated with up to 5 MW RF power and a field gradient of up to 110 MV/m. The temperature of the gun is stabilized to < 10 mK to reduce the gun contribution to the overall timing jitter. The UV laser light for photoemission is coupled-in at a small angle with respect to the beam axis using an in-vacuum mirror located about 0.5 m downstream of the photocathode resulting in spot size of 10 µm (rms). The chosen combination of the laser spot size, cathode material, and field gradient results in a beam emittance of 20 nm (rms).

### S4. Buncher cavity

The buncher is a 4-cell S-band cavity located about 1.2 m downstream of the photocathode RF-gun. The beam passes on the zero-crossing of the field through the cavity so that a correlated energy spread is impinged onto the bunch leading to a longitudinal bunch compression in the following drift section towards the sample. The field amplitude is tuned such that the bunch length reaches a minimum at the sample position.

### S5. REGAE diffraction experiment

#### S5.1. Large experimental chamber

For diffraction experiments REGAE is equipped with a large experimental UHV chamber (Fig. S3). The chamber is internally equipped with 4 heavy-load horizontal translation stages allowing to carry and precisely position experimental equipment in the electron beam path.

#### S5.2. Inline sample viewing microscope

For sample visualization and beamline alignment an inline sample viewing microscope can be inserted into the electron beam path allowing to permanently visualize the sample with optical light also during the diffraction experiment. Its functioning principle is illustrated in Fig. S4. The inline microscope allows to view the sample with visible light colinear to the electron beam. For this, the electron beam passes through a 1.0 mm drill hole along the optical axis of the microscope objective. Shortly after the light exits the microscope it is deflected upwards by a 90-degree deflecting prism, which is equipped with a drill hole. A tube lens then focuses the light on a CCD camera. Non-UHV compatible parts of the device such as the CCD camera are placed in a sealed containment at atmospheric pressure, allowing to operate the device in a UHV environment. The on-axis microscope provides a field of view of 1250 × 1050 µm with an optical resolution of about 1 µm.

The 1.0 mm drill hole in the objective is typically fitted with a tantalum capillary with an inner diameter of 0.5 mm serving as a collimator for the electron beam leading to significant reduction of the background signal on the detector. The entire device is mounted on a motorized 5-axes positioning system allowing for x-, y-, and z-positioning and angular alignment with pitch and yaw. The inline viewing microscope was purchased from suna precision (www.suna-precision.com).

#### S5.3. High-precision $e^-$-Roadrunner crystallography goniometer

For diffraction experiments with solid samples the chamber is equipped with an $e^-$-Roadrunner goniometer, a compact and UHV compatible single-axis crystallographic goniometer with a high-precision vertical rotation axis (Fig. S5). The goniometer axis itself consists of a directly servomotor-driven and UHV compatible rotation axis based on ceramic bearings providing 360-degree rotation capability with an angular resolution of 0.001° in combination with a sphere of confusion smaller than 1 µm. A centring stage mounted on top of the rotation axis provides travel ranges of +/- 6 mm in x-, y-, and z-direction with a resolution < 1 µm and allows precise positioning of the sample in the centre of the

rotation axis. This assures that the sample remains in the electron beam during rotation for data collection. The goniometer axis itself is mounted on a 3-axes motorized translation stage in an orthogonal configuration allowing to position the rotation axis in x-, y-, and z-direction precisely at the position of the electron beam. The goniometer can carry different sample holders ranging from custom made rectangularly shaped silicon nitride membranes on a silicon frame to conventional round 3 mm diameter standard EM grids. The assembly of the on-axis microscope together with the $e^-$ Roadrunner goniometer and a sample holder are shown in Figure S6.

### S5.4. Jungfrau 1M pixel detector

Diffraction images are recorded on a UHV compatible version of the Jungfrau 1M detector, which is placed 5.34 m downstream of the sample and terminating the vacuum system (Fig. S7). The detector has been originally developed for experiments with high-intensity X-ray pulses at X-ray free-electron lasers but can also be used for experiments at synchrotron radiation sources (Mozzanica *et al.*, 2018). The Jungfrau detector directly records the electrical signal generated by the inelastic interactions of the electrons with the silicon sensor material. This signal, which is proportional to the energy deposited in the sensor and given its strong intensity, allows achieving a higher signal over noise ratio than possible with commonly used indirect scintillator-based detection. Each 3.48 MeV electron impinging on the detector surface will behave as a minimum ionizing particle, ionizing e-h+ pairs along its mostly straight track and then exiting the back side of the sensor with most of its energy and momentum. It is then going to release the rest of the energy and stop in the downstream readout, which can withstand radiation doses up to a ~1 MGy, so that radiation damage for operating condition at REGAE is not expected. The Jungfrau 1M detector is composed of two 500 kpixel modules. The pixel size is 75 µm squared and the detector can be operated at frames rates of up to 2 kHz. The system is water-cooled at room temperature, and is operated at vacuum levels $< 3 \times 10^{-7}$ mbar for the measurements.
By using automatic in-pixel gain switching with three different feedback capacitors Jungfrau provides a sufficient dynamic range for detecting strong Bragg reflections while at the same time maintaining single electron resolution with a high signal to noise ratio. In our case with a 320 µm thick silicon sensor the dynamic range is around 1200 incident electrons per pixel and frame (Fröjdh *et al.*, 2022).

## S6. Sample Preparation

### S6.1. Muscovite

The muscovite sample (mica, "rectangular #56 - 75 × 25 mm$^2$", quality "V1") was purchased from Plano GmbH (Germany). To obtain a clean surface the muscovite plate was exfoliated several times with adhesive tape until a flat surface was achieved (Novoselov & Neto, 2012) (Fig. S8). Subsequently, thin multilayer sheets with optically smooth and homogeneous surfaces and without obvious cracks were produced from the muscovite supply plate by exfoliation with adhesive tape again. These multilayer sheets were then glued over the apertures with a size 0.75 × 3.00 mm$^2$ of the REGAE silicon sample holders with dimensions of 5 × 5 mm$^2$ and thickness of 0.2 mm using ethyl-2-

cyanoacrylate adhesive (UHU "mini blitzschnell" gel, UHU GmbH & Co KG; Fig. S6). After curing of the glue, the sample was further thinned down by additional exfoliation so that ideally a very thin sample consisting of few layers only was obtained. The sample used for the diffraction experiment spanned the entire aperture of the silicon sample holder with dimensions of 0.75 × 3.0 $mm^2$ and had a thickness of 670 nm as determined independently with scanning electron microscopy measurements at an edge of the sample. The large aperture size of the sample holder allows for large rotation angles without obstructing the electron beam by the support frame.

### S6.2. Tantalum disulfide (1T-$TaS_2$)

1*T*-$TaS_2$ bulk single crystals were grown from highest purity substrate elements by chemical vapor transport using iodine as transport agent (Novoselov & Neto, 2012). A plate-like single crystal showing well defined crystal edges and a smooth surface showing no major defects with dimensions of about 2 × 2 × 0.2 $mm^3$ was selected. 1*T*-$TaS_2$ crystals exhibit good cleavability into layers perpendicular to the direction of the shortest crystal dimension, the crystallographic c-axis. Very thin sheets of 1*T*-$TaS_2$ with thicknesses down to 25 nm as required for experiments at REGAE were obtained by microtome cutting. For this, the crystal was first glued with epoxy glue onto the top of an epoxy resin block for subsequent microtome cutting (Fig. S9). The sample was then trimmed with a knife to a freestanding and well accessible rectangular shape. The block with the trimmed sample on top was then mounted on the microtome head. Cutting was performed using a LEICA, Ultramicrotome EM UC7 with feed steps of 30 nm and a knife speed of 6 mm $s^{-1}$. Subsequent to slicing, the sample swimming on the water surface was collected with SiN-coated silicon chips and was dried in the air. The sample flake used for the diffraction experiment had lateral dimensions of 0.9 × 0.4 $mm^2$ and a thickness of 30 nm. For the measurements the flake was mounted on a silicon frame equipped with rectangular apertures covered with 30 nm $Si_3N_4$ membranes.

## S7. Data collection

Crystallographic rotation data from the muscovite and 1*T*-$TaS_2$ samples were collected at REGAE facility at room temperature using an electron energy of 3.48 MeV. Before data collection both samples were centred in the goniometer rotation axis and in the electron using the inline-sample viewing microscope. Data collection was performed in step scan mode. For this the sample was first rotated to the starting angle and a predefined number of single shot diffraction images were recorded at this position. Then the sample was rotated by a small increment where again the same predefined number of rotation images was recorded. This procedure was repeated until diffraction images were recorded over the full rotation range. The total accessible rotation range for a given sample is limited by the increasing effective thickness of the sample at higher rotation angles. In our case the overall rotation range was selected such, that for both start and end position still a few diffraction spots were visible on the diffraction images. Data collection parameters of both samples at REGAE are summarized in Tab. S1.

### S7.1. Muscovite

Muscovite diffraction data were collected in step scan mode over a rotation range from -60° to +60°. (dataset name: 231215_mica020) The sample was rotated in angle increments of 0.01°. Per rotation increment 12 images were recorded at a repetition rate of REGAE of 12.5 Hz and a bunch charge of 60 fC. The muscovite sample was spanning over the entire aperture of the sample holder with an opening of 0.75 x 3.00 mm, which was defining the accessible lateral size and had a thickness of 670 nm (as independently determined with scanning electron microscope measurements at an edge of the sample).

### S7.2. Tantalum disulfide (1*T*-$TaS_2$)

$TaS_2$ data were collected in step scan mode over a total rotation range from -65° to +65° (dataset name: 240920_TaS2_b09). The sample was rotated in increments of 0.005°. Per rotation increment diffraction patterns from 12 electron pulses were collected and summed up into a single image after application of gain and pedestal correction. REGAE was operating at a frequency of 12.5 Hz with a charge of < 15 fC per shot during data collection. The $TaS_2$ sample had lateral dimensions of 900 × 400 $\mu m^2$ with a thickness of 30 nm.

## S8. Data processing

The JUNGFRAU detector data are stored in HDF5-format. For few frames, either lines or detector segments were apparently not properly written due to overflows in the data back-end system. In those cases, the erroneous pixel counts were replaced by values determined by interpolation from neighboring pixels of the same frame and/or adjacent frames using self-written Python scripts. Similarly, dead pixels were replaced by interpolated values. Pedestal and gain mode corrections were applied to the individual diffraction images (Redford *et al.*, 2018). To reduce disk space and simplify downstream data handling all images recorded at every specific rotation angle were summed up into one frame after the corrections. Non-detecting detector segments within the detector outline, i.e. the physical borders between detector modules, were masked and ignored for the data reduction. For the estimation of standard uncertainties, a gain of 10 counts per electron was assumed and the variance was set to 30 $counts^2$ (unit: counts squared) corresponding to the PETS2 (Palatinus *et al.*, 2019*b*) input parameter '*noiseparameters 10.0 30.0'*.

### S8.1. Muscovite

To facilitate data processing of the 12800 muscovite diffraction images, 10 images of neighbouring rotation angles were summed up and combined into a single image, which then corresponds to a rotation increment of 0.1°, and exported as unsigned 32-bit TIFF file for subsequent data reduction with PETS2 (Palatinus *et al.*, 2019*b*). Out of this reduced number of 1280 frames from the muscovite diffraction dataset, 1216 frames were (frame numbers 64 to 1279) used for the data reduction.

During the initial peak search, pattern centres were determined by averaging the coordinates of automatically identified Friedel pairs. A fixed unit cell with parameters $a$ = 5.18 Å, $b$ = 8.99 Å, $c$ =20.03

Å, $\alpha = \gamma = 90.0°$, and $\beta = 95.76°$ was used to find the initial orientation matrix (Catti *et al.*, 1994). As expected from tests with polycrystalline samples, a strong elliptical distortion was identified and corrected for using the distortion options in PETS2 (Brázda *et al.*, 2022). The elliptical amplitude refined to 1.57% with a phase angle of 153.6°. An attempted optimisation of the frame orientation angles did not improve the data reduction and thus the nominal orientation angles were used. Finally, only the pattern centres and the frame-dependent optimisation of the magnification was optimised. The pattern centre moved during the experiment, with the $x$ coordinate being 342.4 (9) pixels, and that of the $y$ coordinate 396.3 (7) pixels. The $x$ coordinate oscillates in a sinusoidal way with an amplitude of about 0.75 pixels and a separation between two maxima of about 120 frames, corresponding to an oscillation frequency of about 0.083 Hz. Integrated intensities were determined using a 2D profile fitting routine assuming a rocking curve width of 0.0 Å$^{-1}$ and a mosaicity of 0.4°. For structure solution and kinematical refinement, frame scales were applied assuming that the Laue class is $2/m$ and standard uncertainties of reflection intensities were determined as recommended in Khouchen *et al.* (Khouchen *et al.*, 2023). For the dynamical refinement, geometric parameters and integrated reflections intensities were exported without any correction or scale factors. Reflections were bundled into virtual frames so that 28 processed frames with $\Delta\alpha_v = 2.8°$ make up one virtual frame. Subsequent virtual frames overlap by $\Delta\alpha_o = 1.0°$.

### S8.2. Tantalum disulfide $TaS_2$

Data treatment was performed analogous to the previous section. Again, 30 frames taken at neighbouring rotation angles were summed up for data reduction with PETS2, further reducing the number of frames to be analysed from 26.000 to 866 (Palatinus *et al.*, 2019).

The reflections from the peak search could not be indexed with a conventional lattice with 3 basis vectors. The strongest reflections lie on a hexagonal lattice, and all remaining reflections are satellite reflections that can be indexed using two modulation wave vectors $\mathbf{q_1} = (0.2450, 0.0681, 1/3)$ and $\mathbf{q_2} = (-0.0681, 0.3131, 2/3)$. Integrated intensities were determined by simply adding up the pixel intensities within a circular reflection mask. The reflection positions were predicted assuming a rocking curve width of 0.0025 Å$^{-1}$ and a mosaicity of 0.15°.

For structure solution and kinematical refinement, frame scales were applied assuming that the Laue class is $\bar{3}$ and standard uncertainties of reflection intensities were determined as recommended in Khouchen *et al.* (Khouchen *et al.*, 2023) For the dynamical refinement, geometric parameters and integrated reflections intensities were exported without any corrections. Note that Laue indices consist of five indices *hklmn*. Reflections were bundled into virtual frames so that 20 processed frames with $\Delta\alpha_v = 3.0°$ make up one virtual frame. Subsequent virtual frames overlap by $\Delta\alpha_o = 1.05°$. After the data reduction, in the output files the unit cell parameter $c$, the vector $\mathbf{c^*}$ of the orientation matrix, the modulation wave vectors and the reflection indices *hklmn* were adapted in the output files to achieve a super-centred setting:

$$c' = 3c$$

$$\mathbf{c}^{*\prime} = \frac{1}{3}\mathbf{c}^*$$
$$\mathbf{q}_1' = (0.2453, 0.0679, 0)$$
$$\mathbf{q}_2' = (-0.0679, 0.3132, 0)$$
$$l' = 3l + m + 2n$$

In this setting, the super-space structure can be described in a super-centred setting, where the centring vectors are $(0,0,0,0,0), \left(0,0,\frac{1}{3},\frac{2}{3},\frac{1}{3}\right), \left(0,0,\frac{2}{3},\frac{1}{3},\frac{2}{3}\right)$. Table S2 and Fig. S10 illustrate symmetrical relationships between the 18 modulation wave vectors expressed as linear combinations of $\mathbf{q}_1'$ and $\mathbf{q}_2'$, which are grouped into 6 groups. Note that in all cases satellite reflections with modulation wave vector ($-m$,$-n$) belong to the group ($m$,$n$). The absolute length of $|m\mathbf{q}_1' + n\mathbf{q}_2'|$ is used to order the groups of satellite reflections from lower to higher orders.

### S9. Structure solution and refinement - muscovite

#### S9.1. Structure solution

Based on the output for kinematical refinement, structure solution was successfully attempted with various approaches. Despite the limited completeness of the data set, simple direct methods as implemented in SIR2014 (Burla *et al.*, 2015), charge flipping as implemented in SUPERFLIP (Palatinus & Chapuis, 2007), and a dual-space approach as implemented in shelxt (Sheldrick, 2015) solved the structure (Fig. S11).

#### S9.2. Kinematical refinement

For the subsequent kinematical refinement, it was assumed that the tetrahedral sites are statistically occupied by 25% Al and 75% Si without refining any occupancies. A refinement of site coordinates and isotropic displacement parameters yields $R_{\mathrm{all}}$ of 35.1%. This improves significantly by using an extinction correction based on the EXTI-approach (Sheldrick, 2008).The final refinement with anisotropic displacement parameters converges with $R_{\mathrm{all}}$ of 17.7%. At this stage, the structure model is not charge balanced. In the difference Fourier map, there is one peak at the 2.7$\sigma$ level at a distance of 1.01 Å from the O6 site. The free refinement of a hydrogen atom placed at the peak lowers the $R_{\mathrm{all}}$ to 17.3% and the O-H distance is 1.07 (3) Å based on the kinematical refinement.

#### S9.3. Dynamical refinement

For the dynamical refinement, the respective output file of PETS2 (Palatinus *et al.*, 2019*b*) was loaded into Jana2020 (Petříček *et al.*, 2023) together with the model from the kinematical refinement without extinction correction. The Bloch wave approach is then used to determine calculated intensities and their derivatives with respect to refinement parameters needed for the least-squares refinement. With the default thickness of 40 nm and optimised scales, $R_{\mathrm{all}}$ was 37%. A series of fixed-model refinements tested the dependence of $R_{\mathrm{all}}$ on the crystal thickness in the range between 40 nm and 800 nm, revealing a clear minimum at 600 nm (Fig. 3A). With this value, $R_{\mathrm{all}}$ dropped to 19%. A subsequent

refinement of the model parameters with an increased number of integration steps, $R_{all}$ was further lowered to 14.2%. Finally, the mosaicity of the crystal was modelled as the superposition of incoherently scattering domains representing an isotropic mosaicity of 0.26°. After optimising the orientation angles of the virtual frames, the final refinement with anisotropic displacement parameters converged with $R_{all}$ = 9.9% for 4426 reflections and a refined thickness of 634 (3) nm. Further details on the data set and dynamical refinement are given in Tab. S3.

Apart from the constraints related to Al and Si on tetrahedral sites, no further constraints are used and in the resulting structural model, meaningful anisotropic displacement parameters are obtained (Fig. 3D).

The hydrogen atom present in the structure was initially identified as additional electrostatic potential in the difference Fourier map close to the 3σ level near the O6 site. In our dynamical structure refinements, we were able to freely refine the position of the hydrogen atom together with anisotropic thermal displacement parameters (ADPs). All refined parameters for the hydrogen atom agree very well with the most recent model from single crystal neutron diffraction measurements within a $2\sigma$ limit (Tab. S4) (Gatta *et al.*, 2011). This agreement is also apparent from a visual comparison of the displacement ellipsoids (Fig. 3E).

### S10. Structure solution and refinement - Tantalum disulfide 1*T*-$TaS_2$

#### S10.1. Average structure model

For initial testing purposes, the 3D average structure was determined based on reflections *hkl00*. A Python-script was used to generate an hkl-file with three indices, filtering out satellite reflections. This file was imported with JANA2020. Structure solution in space group $P\bar{3}m$ was straightforward with the three programs used in Section 5.1. A kinematical refinement resulted in very high $R$ factors without ($R_{all}$ = 28.5%) and with extinction correction ($R_{all}$ = 24.9%). A dynamical refinement of the unmodulated structure converged with $R_{all}$ = 5.8% ($MR_{all}$ = 4.3%). In the latter model, the anisotropic displacement parameters of sulphur indicated a pronounced displacement parallel to **c**, whereas the displacement ellipsoid of Ta was rather oblate indicating pronounced displacements perpendicular to **c**.

#### S10.2. Structure solution of the modulated structure

For the determination of the structure in (3+2)d super space, the transformed output file from the data reduction was imported to JANA2020. Reflections were merged based on point group −3 resulting in 11067 unique reflections. The modulated structure was solved with SUPERFLIP (Palatinus & Chapuis, 2007). The resulting 5D map is in agreement with the symmetry of the super space group $X\bar{3}(\alpha,\beta,0)0(-\alpha-\beta,\alpha,0)0$, which is a supercentered setting of the group 147.2.72.1 in the tables by Stokes and Campbell (https://iso.byu.edu/ssg.php) with centring vectors (0,0,0,0,0), (0,0,1/3,2/3,1/3), and (0,0,2/3,1/3,2/3). The solution indicates a strong displacive modulation of the tantalum site within

the *ab*-plane, but no displacive modulation along **c**. For the sulphur site, the displacive modulation is pronounced in the *ab*-plane and along **c**.

### S10.3. Kinematical refinement of the modulated structure

18 modulation waves (Table S2) for six groups of satellite reflections were defined in JANA2020 (Petříček *et al.*, 2023*a*). Based on the structure solution, five parameters for the displacive modulation of the $x$ and $y$ coordinate of Ta were manually set, accounting for the modulation waves of group 1 and group 2. With this approximate starting model, the star-shaped clustering of Ta atoms in 3D sections of the super space model is already recognisable. Subsequently, a kinematical refinement was performed using the constraints derived by symmetry, which are set automatically by JANA2020. At first, parameters for the displacive modulation functions of Ta and S for modulation waves of group 1 and group 2 were refined. Then, displacive modulation parameters belonging to the next-higher order group were refined, until the parameters of all defined modulation waves converged. De Wolff-sections of the refined super space model agree well with sections from the structure solution (Fig. S12). Finally, anisotropic displacement parameters for Ta and S were refined. With extinction correction (EXTI) applied, the final $R_{\text{all}}$ is 24.6% for 9924 reflections. For main reflections, $R_{\text{all}}$ is 21.6%. A refinement with a resolution limit $\frac{\sin\theta}{\lambda} \leq 0.8\ \text{Å}^{-1}$ improves the results with $R_{\text{all}}$ = 19.4% for 4055 reflections.

### S10.4. Dynamical refinement of the modulated structure

The final model of the kinematical refinement was used as starting model for the dynamical refinement. For this purpose, the transformed output file from PETS2 was imported with JANA2020. The resolution limit for reflections used in the refinement was set to $\frac{\sin\theta}{\lambda} \leq 0.8\ \text{Å}^{-1}$ to reduce the computational costs for one refinement cycle. Initial parameters for thickness, scales, and correction parameters for the thickness model were taken from the dynamical refinement of the 3D average structure model. The refinement converged quickly, yielding very promising *R* factors for main reflections and stronger satellite reflections. Modulation parameters of anisotropic displacement parameters were refined, but most of them refined to non-significant values so that only the modulation parameters for U33 of the sulphur site were kept in the refinement. Finally, frame orientation angles were optimised before the final round of refinement cycles. Dataset statistics and dynamical structure refinement parameters for the $TaS_2$ sample are provided in Table S5. Additionally, refinement *R* factors and selected statistics for the individual satellite reflection groups are provided in Table S6.

### S10.5. Equivalent settings

The final structure model can be transformed into several equivalent settings (van Smaalen et al., 2013). For example, a rotation by 180° about c results in a setting where S is located at (1/3, 2/3, z). As indicated in Table S2 and visualised in Figure S10, there are 6 symmetrically equivalent modulation wave vectors for q1, of which 5 are linearly independent. Any two of these 5 are valid choices for

q1 and q2. In this study and in (Spijkerman et al., 1997) with a description in the superspace group $\boldsymbol{X\bar{3}(\alpha,\beta,0)0(-\beta,\alpha+\beta,0)0}$, the relationship between q1 and q2 is the $\bar{\mathbf{3}}$ operation. Stokes et al. (Stokes et al., 2011) define the non-centered standard setting of this superspace group as $\boldsymbol{P\bar{3}\left(\alpha,\beta,\frac{1}{3}\right)0\left(-\alpha-\beta,\alpha,\frac{1}{3}\right)0}$ (number 147.2.72.1), where the operation relating q1 and q2 is that of a 3-fold rotation. For each valid pair of q1 and q2, the structure can be described in a primitive cell or in a super-centered cell. Here, we do not provide a full overview of all possible settings. Instead, we provide the transformation matrices relating the super-centered setting used in this study with the primitive setting and with the settings suggested by Stokes et al. in Table S7.

### S10.6. Comparison with the X-ray based reference model

The resulting model can be directly compared with the one published by Spijkerman et al. (Spijkerman et al., 1997). We noted, however, that inconsistent values of the fractional coordinates of S were given in the reference. This is resolved by assuming that S is found at (1/3, 2/3, −0.0862), which is symmetrically equivalent to (2/3, 1/3, 0.0862). In Tables S8, S9, and S10 the modulation function parameters are compared for Ta at (0, 0, 0) and S at (2/3, 1/3, 0.0862). As defined by Equation 4 in (Spijkerman et al., 1997), modulation functions for $x_i \in \{x, y, z\}$ are described as a Fourier series with Fourier amplitudes $A^{x_i}_{m,n}$ (sine components) and $B^{x_i}_{m,n}$ (cosine components). Note that Spijkerman et al. (Spijkerman et al., 1997) reported the Fourier amplitudes in units of Å, whereas here they are unitless and express the displacement of the fractional coordinates. Also note that the cosine components $B^{x}_{m,n}$, $B^{y}_{m,n}$, $B^{z}_{m,n}$ of Ta are zero due to symmetry constraints.

## S11. Comparative X-ray structure determination of muscovite

### S11.1. Data collection

X-ray diffraction data were recorded from exactly the same crystal as used in our MeV ED experiments before. Data were collected at beamline P11 at the PETRA III synchrotron in Hamburg using an X-ray energy of 20 keV. For data collection the sample was continuously rotated at a constant velocity over an angular range from -60° to +60°. In total 1200 diffraction images corresponding to an oscillation range of 0.1° per image were recorded on an Eiger 16M detector located 152.9 mm behind the sample.

### S11.2. Data analysis and structure refinement

XDS was used for indexing and integration of the X-ray-diffraction data (Kabsch, 2010). Structure refinement using the model from Gatta *et al.* (Gatta *et al.*, 2011) was conducted with OLEX2 (Bourhis *et al.*, 2015) (Supporting Data S3). A summary of the X-ray data collection and structure refinement parameter is provided in Tab. S11.

A comparison of the two structures obtained in this work with high-energy MeV ED and X-ray diffraction with neutron data from Gatta *el al.* (Gatta *et al.*, 2011) performed with the Bilbao crystallographic server (Flor *et al.*, 2016) is provided in Tab. S8. Whereas the atomic coordinates derived from the ED and X-ray measurements in this work from exactly the same sample agree very well for the non-hydrogen atoms with an average deviation of 0.0094 Å, a slightly larger value of 0.032 Å is obtained for the comparison of our MeV ED data with the neutron reference data. These differences in atomic positions most probably occur from a slightly different composition of the two samples. Whereas we were using synthetically generated muscovite, the sample investigated by Gatta *et al.* (Gatta *et al.*, 2011) was naturally occurring muscovite containing mainly iron and other trace metals as impurities giving rise to slightly different atom positions.

### S12. Supporting figures

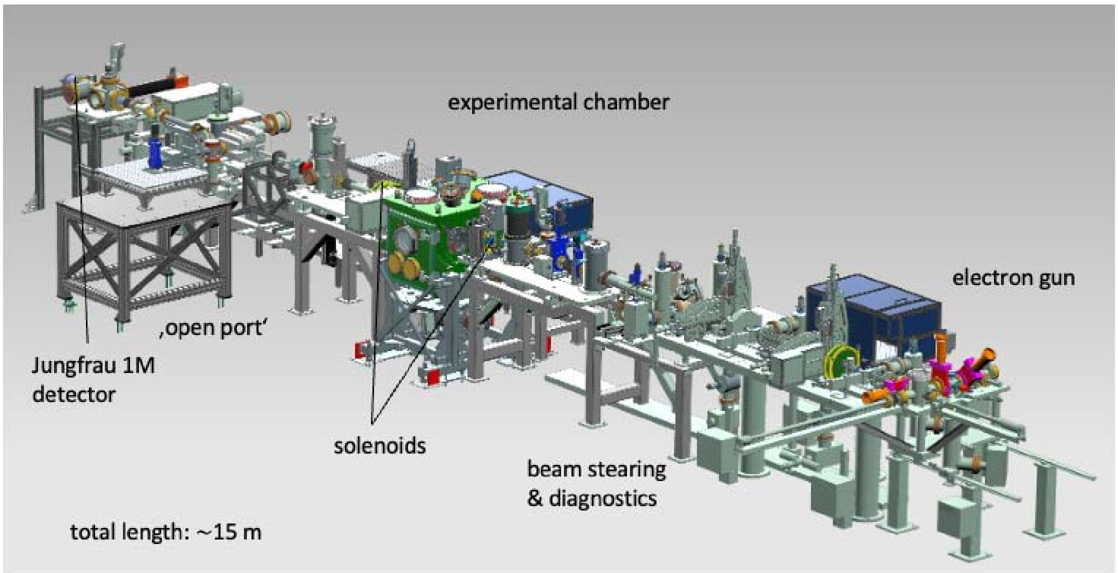


**Figure S1** Technical overview drawing of the REGAE diffraction facility. Electrons are emitted from a photo-cathode (*right side*) and immediately accelerated in a radio-frequency (RF)-cavity to their final energy of 3-5 MeV. A solenoid located shortly before the experimental chamber allows the electron beam to be focused at the interaction point in the experimental chamber, where the sample is located. Diffraction patterns are recorded on a Jungfrau 1M detector (*left side*) capable of direct electron detection, terminating the experimental setup.

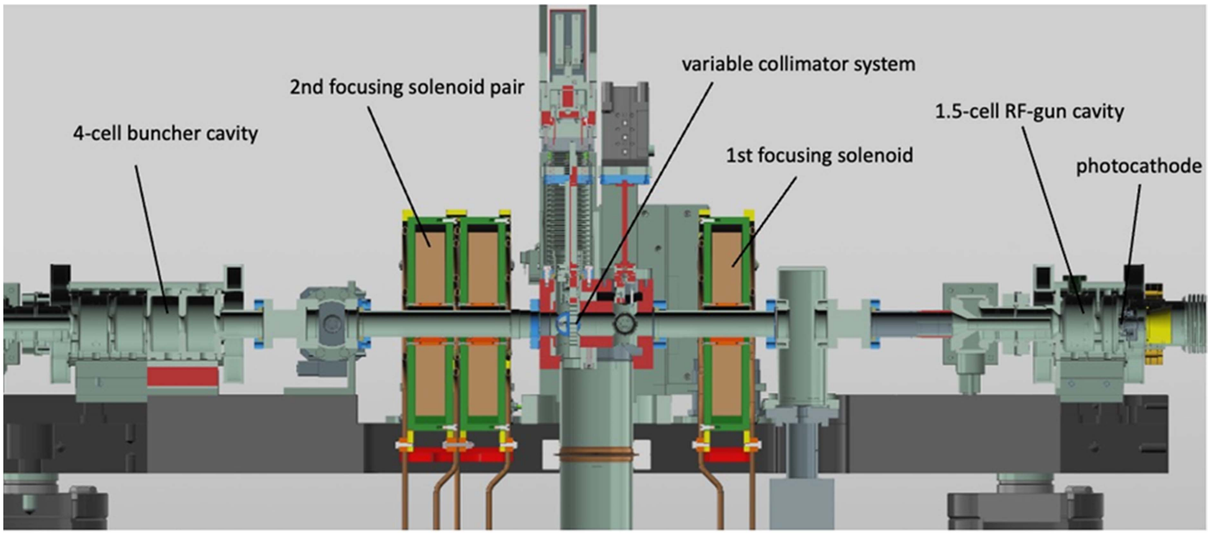


**Figure S2** Detailed view of the REGAE accelerator. Technical drawing of the REAGE accelerator part (*side view*) with labelled key components.

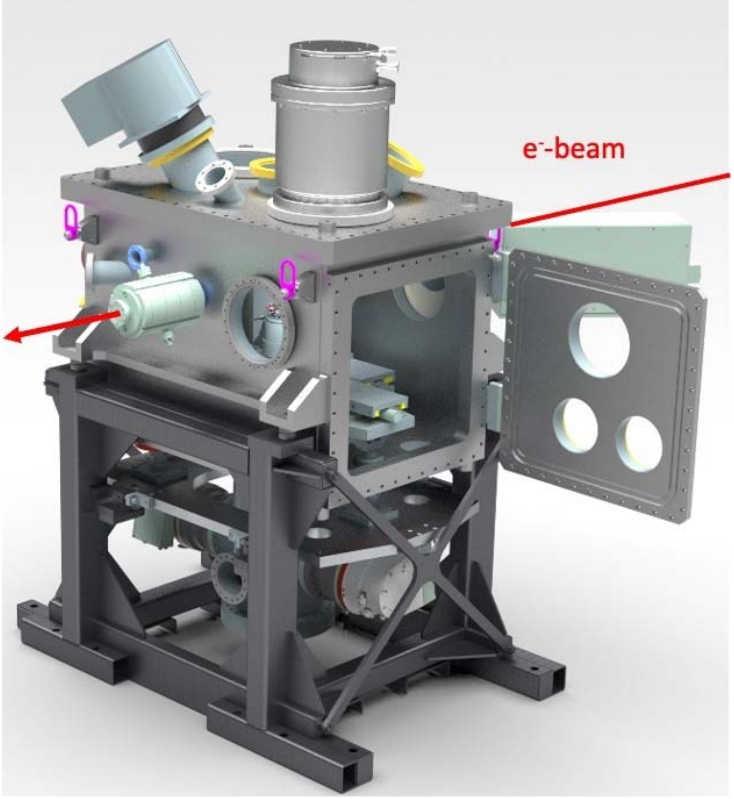


**Figure S3** Large experimental UHV chamber with inner dimensions of 650 × 590 × 960 mm$^3$ for housing different experiments. A large front door allows easy exchange of and access to the equipment. The electron beam enters the chamber on the right side of the chamber and exits on the left.

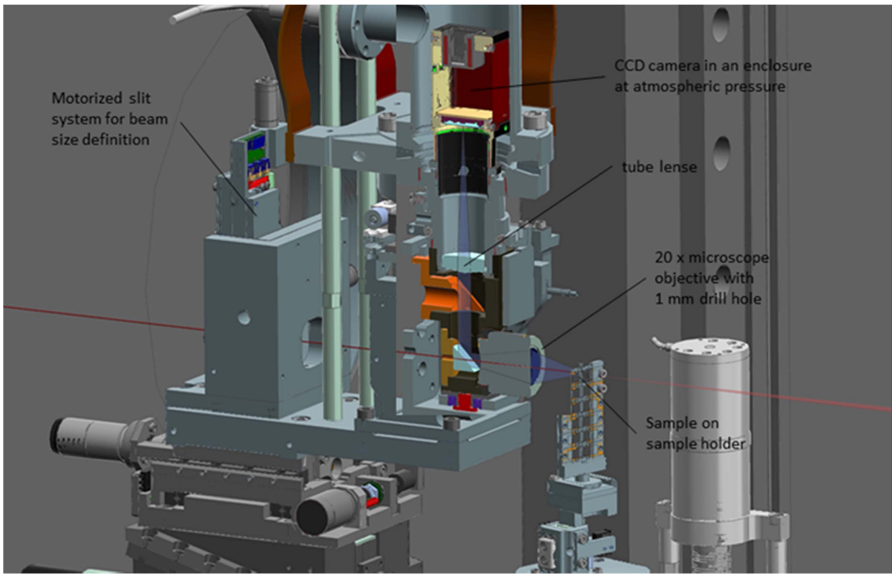


**Figure S4** Functioning principle of the UHV compatible inline sample viewing microscope at REGAE. The electron beam passing through the microscope objective is indicated in red, the visible light coming from the sample is deflected upwards and is then recorded on a CCD camera, indicated in blue. An additional optical port allows in-coupling of laser light for pump-probe experiments with the through-the-lens sample excitation either via an optical fibre or by directly guiding the laser light into the optical port.

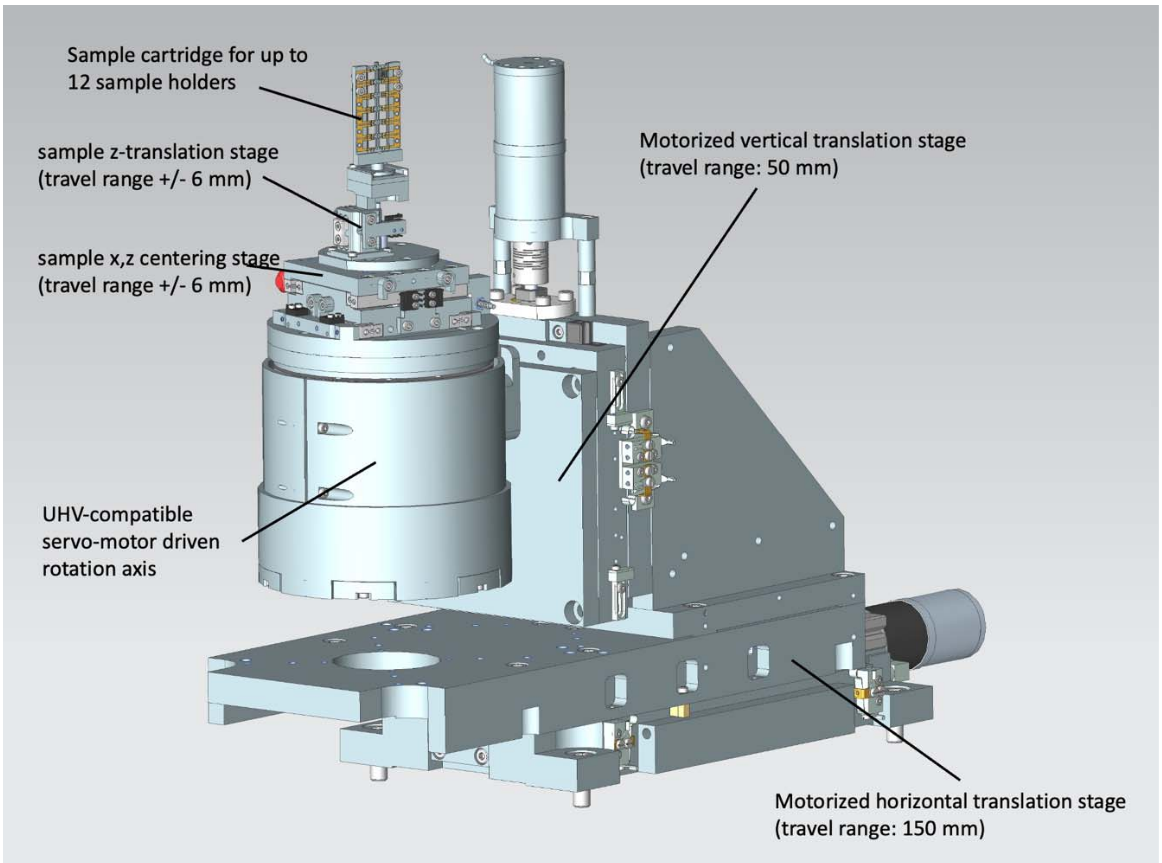


**Figure S5** Technical drawing of the UHV compatible $e^-$-Roadrunner goniometer installed in the REGAE experimental chamber. The rotation axis is oriented vertically and allows for full 360-degree rotation of the sample with an angular resolution of 0.001°. A centring stage mounted on top of the rotation axis allows precise positioning of the sample in the centre of the rotation axis.

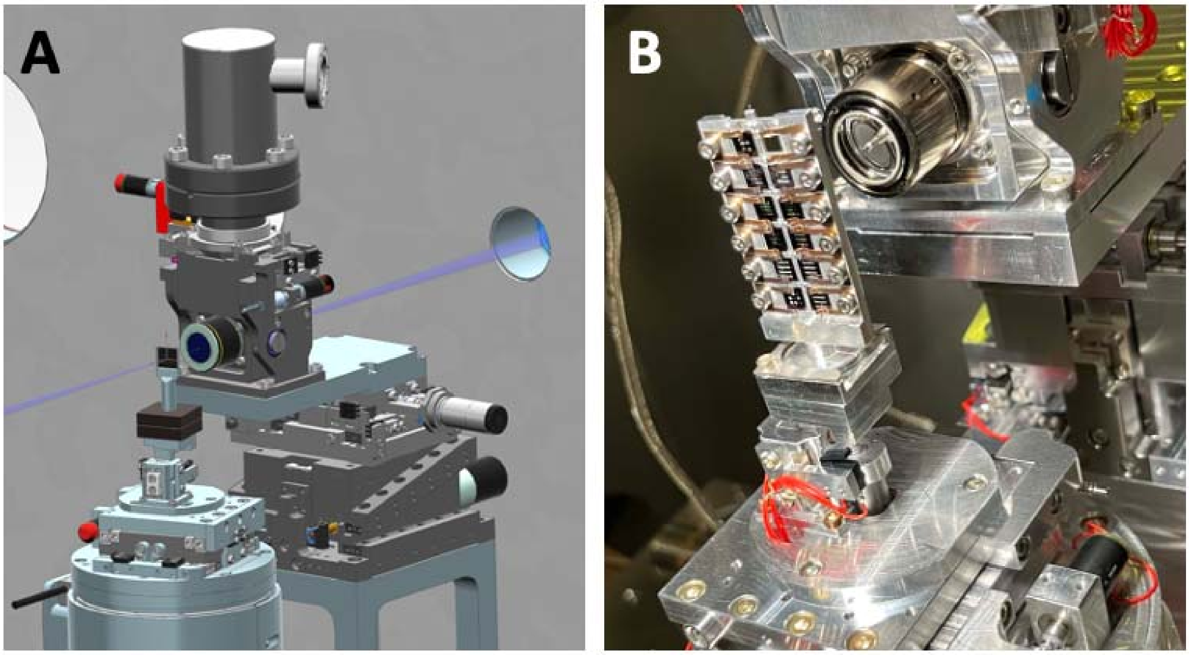


**Figure S6** Technical drawing and photograph of the UHV-crystallography setup installed at REGAE. (**A**) The electron beam (*indicated in blue*) passes first through the drill hole of the on-axis microscope before it is illuminating the sample located on the crystallography goniometer. (**B**) Photograph of sample holder at REGAE mounted on the goniometer and loaded with 12 different samples.

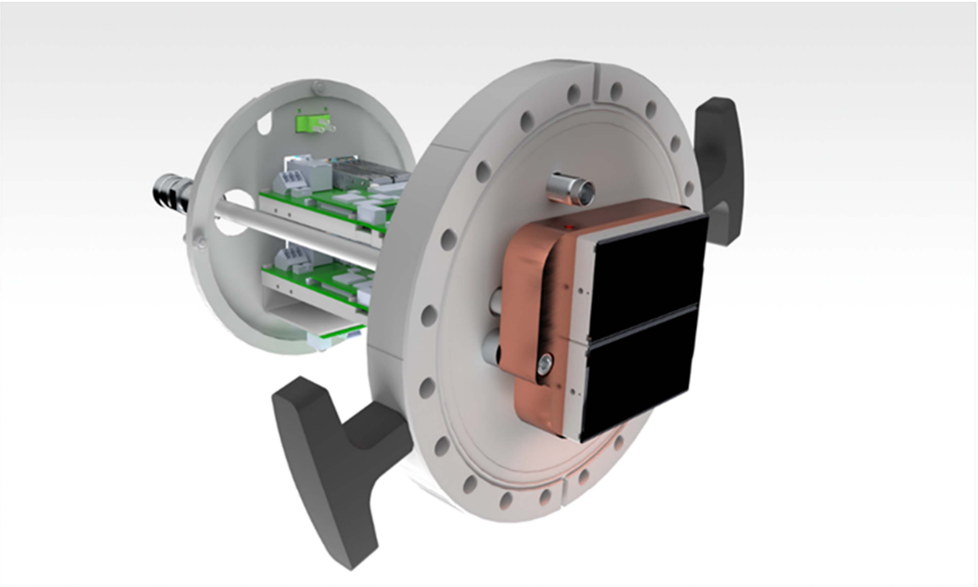

**Figure S7** Jungfrau detector at REGAE. UHV compatible Jungfrau detector installed 5.43 m downstream of the sample for direct recording of the MeV single shot diffraction patterns. The detector consists of two modules of each 500 kPixel with a pixel size of 75 µm with a horizontal gap of 3 mm between the two modules.

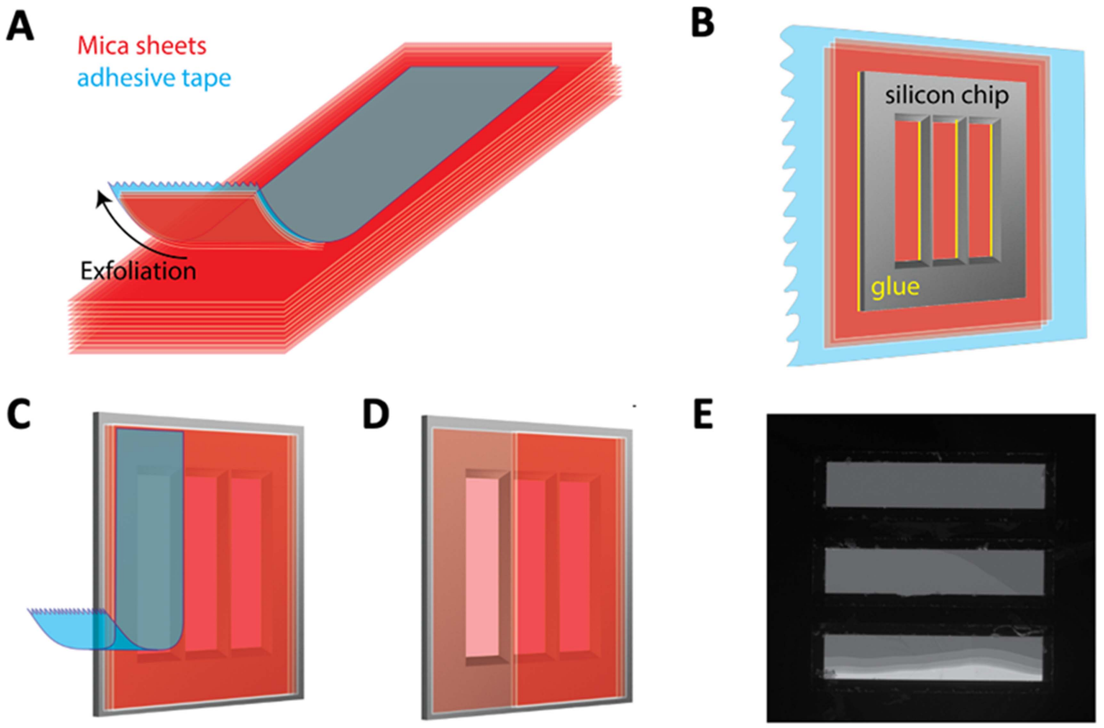


**Figure S8** Muscovite sample preparation using the technique of exfoliation. (**A**) a thin sheet consisting of several layers of muscovite is removed from the bulk crystal. (**B**) This thin sheet is then glued onto the REGAE sample holder. (**C**) The sample thickness is further iteratively reduced by removing layers by exfoliation. (**D**) Sample holder with a thinned down sample covering the left of the three rectangular apertures. (**E**) Micrograph of a thinned-down muscovite sample on a REGAE sample frame manufactured from single crystalline silicon.

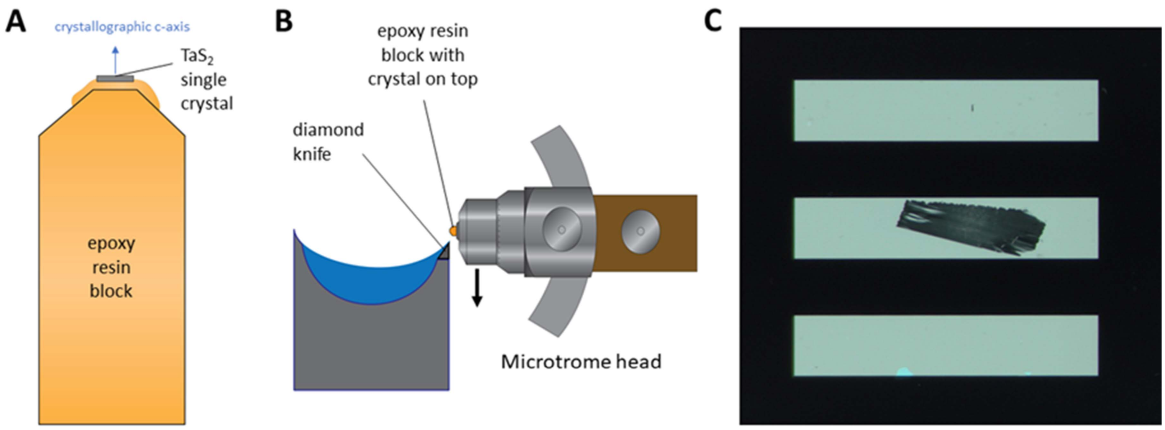


**Figure S9** 1*T*-$TaS_2$ sample preparation using microtome cutting. (**A**) A 1*T*-$TaS_2$ single crystal is glued on top of an epoxy resin block, so that the c-axis (perpendicular to the 1*T*-$TaS_2$ layers) is oriented along the long dimension of the resin block. (**B**) The resin block is inserted into the microtome head. For cutting the microtome head moves downwards so that a thin slice (flake) of the sample is cut off or in this case is cleaved off from the supply crystal with a sharp diamond knife. The thin slices / flakes with adjustable thickness are collected in a water bath. (**C**) A 1*T*-$TaS_2$ flake has been 'fished' from the water bath and is mounted on a REGAE sample holder for the diffraction experiment.

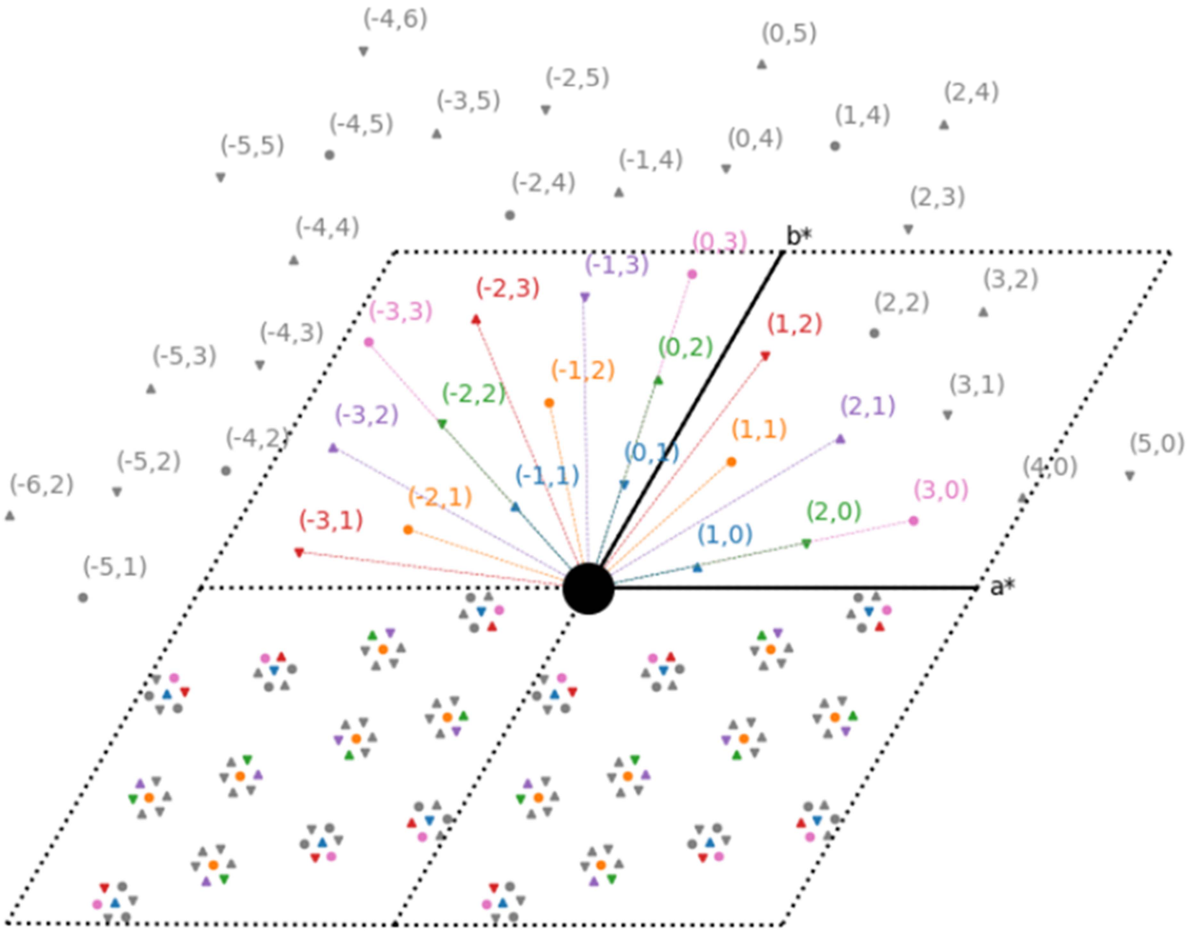


**Figure S10** Indexing scheme for satellite reflections. In the upper part, the positional relationship between main reflections *hkl*00 and satellite reflections *hklmn* is illustrated. Indices (*m*, *n*) of satellite reflections are provided. In the lower part, satellite reflections from neighbouring main reflections are shown. Circles indicate reflections that are present if the condition l = 3n is fulfilled. Triangles pointing upwards are present if the condition l = 3n + 1 is fulfilled. Triangles pointing downwards are present if the condition l = 3n + 2 is fulfilled. Gray satellite reflections are higher-order satellites that are not observed in the data set and not used in the structure analysis. Coloured satellite reflections were used for the structure solution and refinement. Coloured satellites of the same colour belong to the same satellite reflection group. Figure inspired by Figure 2 in Spijkerman *et al.* (Spijkerman *et al.*, 1997).

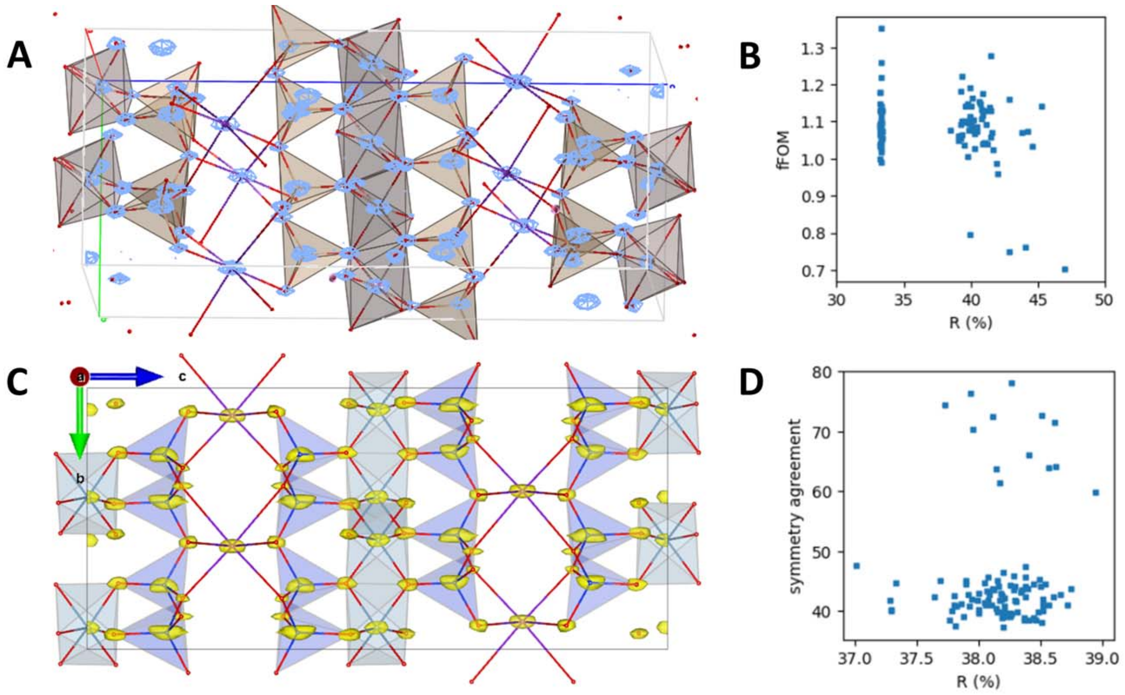


**Figure S11** Ab-initio structure solution of muscovite from REGAE electron diffraction data. (**A**) Electrostatic potential map determined with direct methods using SIR2014. A model is overlaid to visualize the structural interpretation of the distribution of peaks. (**B**) Scatter plot, where each data point represents one solution attempt. Out of 100 attempts, 41 solutions have an R factor below 35% and all checked cases correspond to the correct solution. Higher fFOM values are better. (**C**) Electrostatic potential map determined with charge flipping using SUPERFLIP and visualized using VESTA3. A model is overlaid to visualize the structural interpretation of the distribution of peaks. (**D**) Scatter plot, where each data point represents one solution attempt. Lower symmetry agreement factors are better. Almost all solutions with a symmetry agreement factor below 45 provided useful starting models.

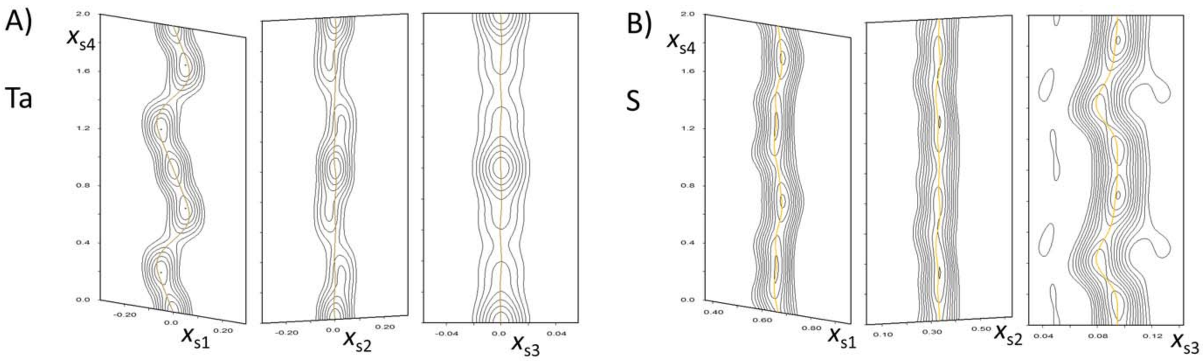


**Figure S12** The modulated structure of 1*T*-$TaS_2$. De Wolff-sections of (**A**) Ta and (**B**) S site with maps from ab initio structure solution and overlaid atomic surfaces from kinematical refinement. Contour lines shown in steps of 33 e/Å (**A**) and 3.3 e/Å (**B**) for Ta and S, respectively. The maximum electrostatic potential of Ta is about 260 e/Å, and that of S is about 25 e/Å. $x_{s5}$ was kept at 0 for all sections, so that these sections only represent a small part of the complete structure solution. Animated de Wolff-sections are provided as supporting movies S1-S6.

### S13. Supporting tables

**Table S1** High-energy electron diffraction data collection parameters of muscovite and $1T$-$TaS_2$ samples.

| **parameter** | **muscovite** | **$1T$-$TaS_2$** |
|---|---|---|
| dataset name | 231215_mica020 | 240920_TaS2_b09 |
| electron energy [MeV] | 3.48(4) | 3.48(4) |
| electron beam diameter [μm] | 250# | 50* |
| pulse charge [fC] | 60 | <15 |
| pulse duration [fs] | ~600 | ~600 |
| rotation range [°] | -60 to +60 | -65 to +65 |
| rotation increment [°] | 0.01 | 0.005 |
| frames per rotation increment | 12 | 12 |
| total number of images | 153.600 | 312.000 |
| number of summed-up diffraction images per rotation increment | 12.800 | 26.000 |
| reduced number of summed up diffraction images for processing with PETS2 (resulting pseudo-rotation range per image) | 1216 (0.1°) | 866 (0.3°) |

# Diameter of the full beam (4σ)

*Defined by a pinhole 100 mm upstream of the sample

**Table S2** Groups of symmetrically related modulation wave vectors.

| **Group ID** | **Group ($m$,$n$)** | **symmetrically related wave vectors #1** | **#2** | **#3** | $\|m\mathbf{q}'_1 + n\mathbf{q}'_2\|$ |
|---|---|---|---|---|---|
| 1 | (1,0) | $1\mathbf{q}'_1$ | $1\mathbf{q}'_2$ | $-1\mathbf{q}'_1 + 1\mathbf{q}'_2$ | 0.10 Å$^{-1}$ |
| 2 | (1,1) | $1\mathbf{q}'_1 + 1\mathbf{q}'_2$ | $-1\mathbf{q}'_1 + 2\mathbf{q}'_2$ | $-2\mathbf{q}'_1 + 1\mathbf{q}'_2$ | 0.17 Å$^{-1}$ |
| 3 | (2,0) | $2\mathbf{q}'_1$ | $2\mathbf{q}'_2$ | $-2\mathbf{q}'_1 + 2\mathbf{q}'_2$ | 0.20 Å$^{-1}$ |
| 4 | (1,2) | $1\mathbf{q}'_1 + 2\mathbf{q}'_2$ | $-2\mathbf{q}'_1 + 3\mathbf{q}'_2$ | $-3\mathbf{q}'_1 + 1\mathbf{q}'_2$ | 0.26 Å$^{-1}$ |
| 5 | (2,1) | $2\mathbf{q}'_1 + 1\mathbf{q}'_2$ | $-1\mathbf{q}'_1 + 3\mathbf{q}'_2$ | $-3\mathbf{q}'_1 + 2\mathbf{q}'_2$ | 0.26 Å$^{-1}$ |
| 6 | (3,0) | $3\mathbf{q}'_1$ | $3\mathbf{q}'_2$ | $-3\mathbf{q}'_1 + 3\mathbf{q}'_2$ | 0.29 Å$^{-1}$ |

**Table S3** Dataset statistics and dynamical structure refinement parameters for the muscovite sample investigated with the method of high-energy electron diffraction.

| | |
|---|---|
| **Crystal / Sample** | |
| Chemical sum formula | $KAl_3Si_3O_{10}(OH)_2$ |
| *Z* | 4 |
| Crystal system | monoclinic |
| Space group | *C*2/*c* |
| *a*, *b*, *c* (Å) | 5.2037(10), 9.0305(18), 20.156(4) |
| *α*, *β*, *γ* (deg.) | 90, 95.78(3), 90 |
| *V* (Å³) | 942.3(3) |
| **Data collection** | |
| Diffractometer | REGAE, DESY, Hamburg |
| Acceleration (kV), *λ* (Å) | 3480, 0.0031 |
| Detector | Jungfrau 1M |
| Sample temperature (K) | 293 |
| Rotation range $\alpha_{min}$, $\alpha_{max}$, $\Delta\alpha_{step}$ (deg.) | −60.0, +60.0, 0.01 |
| Rotation range per saved frame (deg.) | 0.1 |
| $h_{min}$, $h_{max}$ | −9, 8 |
| $k_{min}$, $k_{max}$ | −13, 15 |
| $l_{min}$, $l_{max}$ | −28, 28 |
| Mosaicity (PETS2) (°) | 0.4 |
| **Data set statistics** | |
| High-resolution limit (Å) | 0.56 |
| Completeness (%) | 66.5 |
| Multiplicity | 2.4 |
| $<I/\sigma>$ (based on counting statistics) | 16.1 |
| Measured reflections | 4608 |
| Unique reflections | 1968 |
| Unique reflections $I > 3\sigma$ | 1945 |
| **Dynamical refinement** | |
| virtual frames $\Delta\alpha_v$, $\Delta\alpha_o$ (°) | 2.8, 1.0 |
| No. of scale parameters | 67 (one per virtual frame) |
| No. of structural parameters | 96 |
| No. of refinement parameters | 164 |
| Reflection selection $RSg_{max}$, $DSg_{min}$ (Å$^{-1}$) | 0.67, 0.005 |
| Bloch waves $g_{max}$ (Å$^{-1}$), $Sg_{max}$ (Å$^{-1}$) | 2.5, 0.01 |
| Refined thickness (nm) | 634 (3) |
| Reflections used in refinement $N_{obs}$, $N_{all}$ | 4120, 4426 |
| $R_{obs}$, $R_{all}$, $wR_{all}$ | 9.8%, 9.9%, 10.8% |
| Post-refinement merged $MN_{obs}$, $MN_{all}$ | 1756, 1874 |
| $MR_{obs}$, $MR_{all}$, $MwR_{all}$ | 9.2%, 9.4%, 10.0% |

**Table S4** Comparison of the atomic coordinates and anisotropic thermal displacement parameters for the hydrogen atom of the muscovite structure obtained with MeV ED in our work with reference data from neutron diffraction (ND) experiments by Gatta *et al.* (Gatta *et al.*, 2011).

| Source | T (K) | fractional atomic coordinates | | | | | |
|---|---|---|---|---|---|---|---|
| | | x | | y | | z | |
| ED (this work) | 293 | 0.129(6) | | 0.1508(26) | | 0.4387(28) | |
| ND (Gatta *et al.*, 2011) | 295 | 0.1311(7) | | 0.1527(4) | | 0.4420(3) | |
| $\Delta=\lvert p_1 - p_2\rvert$ | | $0.5\sigma_\Delta$ | | $0.7\sigma_\Delta$ | | $1.2\sigma_\Delta$ | |
| | | **anisotropic thermal displacement parameters (ADPs)** | | | | | |
| | | $U_{11}$ (Å$^2$) | $U_{22}$ (Å$^2$) | $U_{33}$ (Å$^2$) | $U_{12}$ (Å$^2$) | $U_{13}$ (Å$^2$) | $U_{23}$ (Å$^2$) |
| ED (this work) | 293 | 0.068(21) | 0.032(12) | 0.17(7) | −0.009(1 | 0.019(28) | 0.052(19) |
| ND (Gatta *et al.*, 2011) | 295 | 0.035(2) | 0.034(2) | 0.085(3) | −0.002(1) | 0.018(2) | 0.021(2) |
| $\Delta=\lvert p_1 - p_2\rvert$ | | $1.6\sigma_\Delta$ | $0.2\sigma_\Delta$ | $1.2\sigma_\Delta$ | $0.7\sigma_\Delta$ | $0.0\sigma_\Delta$ | $1.6\sigma_\Delta$ |

**Table S5** Dataset statistics and dynamical structure refinement parameters for the $TaS_2$ sample investigated with the method of high-energy electron diffraction.

| | | | |
|---|---|---|---|
| **Crystal / Sample** | | | |
| Chemical sum formula | $TaS_2$ | | |
| $Z$ | 3 | | |
| Crystal system | Trigonal | | |
| Superspace group | $X\bar{3}(\alpha,\beta,0)0(-\alpha-\beta,\alpha,0)0$ | | |
| $a$, $b$, $c$ (Å) | 3.3553 (4), 3.3553 (4), 17.6475 (24) | | |
| $\alpha$, $\beta$, $\gamma$ (deg.) | 90, 90, 120 | | |
| $V$ (Å$^3$) | 127.06 (4) | | |
| $\mathbf{q}_1$, $\mathbf{q}_2$ | [0.2453 (2), 0.0679 (3), 0], [−0.0679 (2), 0.3132 (3), 0] | | |
| **Data collection** | | | |
| Diffractometer | REGAE, DESY, Hamburg | | |
| Acceleration (kV), $\lambda$ (Å) | 3480, 0.0031 | | |
| Detector | Jungfrau 1M | | |
| Sample temperature (K) | 293 | | |
| Rotation range $\alpha_{min}$, $\alpha_{max}$, $\Delta\alpha_{step}$ (deg.) | −65.0, +65.0, 0.005 | | |
| Rotation range per merged frame (deg.) | 0.15 | | |
| $h_{min}$, $h_{max}$ | −8, 8 | | |
| $k_{min}$, $k_{max}$ | −7, 8 | | |
| $l_{min}$, $l_{max}$ | −31, 29 | | |
| $m_{min}$, $m_{max}$ | −3, 3 | | |
| $n_{min}$, $n_{max}$ | −3, 3 | | |
| Mosaicity (PETS2) (°) | 0.15 | | |
| **Data set statistics** | overall | main refl. | satellite refl. |
| High-resolution limit (Å) | | 0.45 | |
| Completeness (%) | | 62.4 | |
| Multiplicity | | 3.16 | |
| <$I/\sigma$> (based on counting statistics) | 18.6 | 189.7 | 14.2 |
| Measured reflections | 34958 | 867 | 34091 |
| Unique reflections | 11069 | 296 | 10773 |
| Unique reflections $I > 3\sigma$ | 6393 | 295 | 6098 |
| **Dynamical refinement** | | | |
| High-resolution limit (Å) | 0.625 | | |
| virtual frames $\Delta\alpha_v$, $\Delta\alpha_o$ (°) | 3.0, 1.05 | | |
| No. of virtual frames | 63 | | |
| No. of scale parameters | 63 | | |
| No. of thickness-related parameters | 3 | | |
| No. of structural parameters | 69 | | |
| No. of refinement parameters | 135 | | |
| Reflection selection $RSg_{max}$, $DSg_{min}$ (Å$^{-1}$) | 0.66, 0.0015 | | |
| Bloch waves $g_{max}$ (Å$^{-1}$), $Sg_{max}$ (Å$^{-1}$) | 1.9, 0.01 | | |
| Reflections used in refinement $N_{obs}$, $N_{all}$ | 20621, 11910 | | |
| $R_{obs}$, $R_{all}$, $wR_{all}$ | 10.0%, 15.2%, 5.3% | | |
| Post-refinement merged $MN_{obs}$, $MN_{all}$ | 3435, 4951 | | |
| $MR_{obs}$, $MR_{all}$, $MwR_{all}$ | 8.9%, 12.4%, 5.5% | | |

**Table S6** Refinement R factors and selected statistics grouped by satellite reflection group. Group (0,0) corresponds to the main reflections. <*I/σ*> is the mean value of the intensity to uncertainty ratios of $N_{all}$ reflections.

| group (*m*,*n*) | <*I/σ*> | $N_{obs}$ | $N_{all}$ | $R_{obs}$ | $R_{all}$ | $wR_{all}$ | $MN_{obs}$ | $MN_{all}$ | $MR_{obs}$ | $MR_{all}$ | $MwR_{all}$ |
|---|---|---|---|---|---|---|---|---|---|---|---|
| (0,0) | 239.4 | 589 | 589 | 4.9% | 4.9% | 5.7% | 136 | 136 | 3.0% | 3.0% | 3.9% |
| (1,0) | 50.1 | 3249 | 3335 | 8.3% | 8.4% | 8.2% | 797 | 804 | 5.9% | 5.9% | 5.2% |
| (1,1) | 16.5 | 2773 | 3400 | 9.6% | 10.6% | 8.9% | 726 | 796 | 7.0% | 7.5% | 5.9% |
| (2,0) | 7.7 | 2113 | 3289 | 11.4% | 14.6% | 11.6% | 636 | 799 | 8.9% | 10.5% | 8.1% |
| (1,2) | 5.6 | 1569 | 3349 | 15.4% | 23.2% | 16.7% | 518 | 807 | 14.0% | 19.4% | 12.6% |
| (2,1) | 2.3 | 898 | 3253 | 28.6% | 40.0% | 33.2% | 343 | 793 | 29.1% | 35.8% | 31.9% |
| (3,0) | 2.0 | 724 | 3414 | 38.2% | 80.8% | 40.4% | 274 | 811 | 47.0% | 80.0% | 40.0% |

**Table S7** Selected equivalent settings of the reported structure of $TaS_2$. The transformation matrix relates the basis of the reported model with the new basis as determined with the tool *TRANS-FORMSSG* (Version 1.5.1, https://iso.byu.edu/transformssg.php).

| Transformation to | Transformation matrix $S^{-1}$ | Modulation wave vectors | Superspace group | Centring vectors |
|---|---|---|---|---|
| **setting used in this study** | $\begin{pmatrix}1&0&0&0&0\\0&1&0&0&0\\0&0&1&0&0\\0&0&0&1&0\\0&0&0&0&1\end{pmatrix}$ | $\mathbf{q}_1' = (\alpha,\beta,0)$ <br> $\mathbf{q}_2' = (-\beta,\alpha+\beta,0)$ | $X\bar{3}(\alpha,\beta,0)0(-\beta,\alpha+\beta,0)0$ | (0,0,0,0,0) <br> $(0,0,\frac{1}{3},\frac{2}{3},\frac{1}{3})$ <br> $(0,0,\frac{2}{3},\frac{1}{3},\frac{2}{3})$ |
| **primitive setting** | $\begin{pmatrix}1&0&0&0&0\\0&1&0&0&0\\0&0&\frac{1}{3}&0&0\\0&0&-\frac{1}{3}&1&0\\0&0&\frac{2}{3}&0&1\end{pmatrix}$ | $\mathbf{q}_1'' = \left(\alpha,\beta,\frac{1}{3}\right)$ <br> $\mathbf{q}_2'' = \left(-\beta,\alpha+\beta,\frac{2}{3}\right)$ | $P\bar{3}\left(\alpha,\beta,\frac{1}{3}\right)0\left(-\beta,\alpha+\beta,\frac{2}{3}\right)0$ | (0,0,0,0,0) |
| **supercentred setting by Stokes *et al.*** | $\begin{pmatrix}1&0&0&0&0\\0&1&0&0&0\\0&0&1&0&0\\0&0&0&1&0\\0&0&0&1&1\end{pmatrix}$ | $\mathbf{q}_1'' = (\alpha,\beta,0)$ <br> $\mathbf{q}_2'' = (-\alpha-\beta,\alpha,0)$ | $X\bar{3}(\alpha,\beta,0)0(-\alpha-\beta,\alpha,0)0$ | (0,0,0,0,0) <br> $(0,0,\frac{1}{3},\frac{2}{3},\frac{2}{3})$ <br> $(0,0,\frac{2}{3},\frac{1}{3},\frac{1}{3})$ |

| | | | | |
|---|---|---|---|---|
| **primitive setting by Stokes *et al.*** | $\begin{pmatrix} 1 & 0 & 0 & 0 & 0 \\ 0 & 1 & 0 & 0 & 0 \\ 0 & 0 & \frac{1}{3} & 0 & 0 \\ 0 & 0 & -\frac{1}{3} & 1 & 0 \\ 0 & 0 & -\frac{2}{3} & 1 & 1 \end{pmatrix}$ | $\mathbf{q}_1'' = \left(\alpha, \beta, \frac{1}{3}\right)$ $\mathbf{q}_2'' = \left(-\alpha-\beta, \alpha, \frac{1}{3}\right)$ | $P\bar{3}\left(\alpha, \beta, \frac{1}{3}\right)0\left(-\alpha-\beta, \alpha, \frac{1}{3}\right)0$ | (0,0,0,0,0) |

**Table S8** Fourier series amplitudes of the sine components ($A^x_{m,n}$, $A^y_{m,n}$, $A^z_{m,n}$) of the displacive modulation function of the Ta site (0,0,0). Empty fields were not refined in this study because the associated satellite reflections were not observed in the experiment.

| | Spijkerman et al. (1997) | | | this study | | |
|---|---|---|---|---|---|---|
| **(m, n)** | $A^x_{m,n}$ | $A^y_{m,n}$ | $A^z_{m,n}$ | $A^x_{m,n}$ | $A^y_{m,n}$ | $A^z_{m,n}$ |
| **(1,0)** | −0.0405 (1) | −0.0201 | 0.0002 | −0.04015 (5) | −0.01905 (5) | 0.000173 (16) |
| **(0,1)** | −0.0204 (1) | −0.0405 | −0.0002 | −0.02110 (7) | −0.04015 (5) | −0.000173 (16) |
| **(−1,1)** | 0.0201 | −0.0204 | 0.0002 (1) | −0.01905 (5) | 0.02110 (7) | −0.000173 (16) |
| **(1,1)** | 0.0170 (1) | −0.0016 | 0.0000 | 0.01737 (7) | −0.00181 (8) | 0.00004 (2) |
| **(−1,2)** | 0.0185 (1) | 0.0170 | 0.0000 | 0.01918 (10) | 0.01737 (7) | −0.00004 (2) |
| **(−2,1)** | 0.0016 | 0.0185 | 0.0000 (1) | 0.00181 (8) | 0.01918 (10) | 0.00004 (2) |
| **(2,0)** | 0.0036 (1) | 0.0137 | 0.0001 | 0.00299 (9) | 0.01397 (7) | 0.00018 (3) |
| **(0,2)** | −0.0101 (1) | 0.0036 | −0.0001 | −0.01098 (11) | 0.00299 (9) | −0.00018 (3) |
| **(−2,2)** | −0.0137 | −0.0101 | 0.0001 (1) | −0.01397 (7) | −0.01098 (11) | 0.00018 (3) |
| **(1,2)** | 0.0000 (2) | 0.0084 | −0.0002 | 0.00093 (13) | 0.00946 (11) | −0.00022 (4) |
| **(−2,3)** | −0.0083 (2) | 0.0000 | 0.0002 | −0.00854 (17) | 0.00093 (13) | 0.00022 (4) |
| **(−3,1)** | −0.0084 | −0.0083 | −0.0002 (1) | −0.00946 (11) | −0.00854 (17) | −0.00022 (4) |
| **(2,1)** | −0.0067 (2) | −0.0064 | 0.0001 | −0.00718 (11) | −0.00723 (12) | −0.00005 (5) |
| **(−1,3)** | −0.0004 (2) | −0.0069 | −0.0001 | 0.00005 (17) | −0.00718 (11) | 0.00005 (5) |

| | | | | | | |
|---|---|---|---|---|---|---|
| **(−3,2)** | 0.0064 | −0.0004 | 0.0001 (1) | 0.00723 (12) | 0.00005 (17) | −0.00005 (5) |
| **(3,0)** | −0.0066 (4) | −0.0115 | −0.0001 | −0.00014 (19) | −0.00691 (17) | 0.00006 (6) |
| **(0,3)** | 0.0049 (3) | −0.0066 | 0.0001 | 0.0068 (3) | −0.00014 (19) | −0.00006 (6) |
| **(−3,3)** | 0.0115 | 0.0049 | −0.0001 (1) | 0.00691 (17) | 0.0068 (3) | 0.00006 (6) |
| **(4,0)** | 0.0119 (6) | 0.0110 | 0.0008 | | | |
| **(0,4)** | 0.0008 (6) | 0.0119 | −0.0008 | | | |
| **(−4,4)** | −0.0110 | 0.0008 | 0.0008 (1) | | | |

**Table S9** Fourier series amplitudes of the sine components ($A^x_{m,n}$, $A^y_{m,n}$, $A^z_{m,n}$) of the displacive modulation function for the S site (2/3, 1/3, 0.0862). Empty fields were not refined in the reference model.

| | **Spijkerman *et al.* (1997)** | | | **this study** | | |
|---|---|---|---|---|---|---|
| ***(m, n)*** | $A^x_{m,n}$ | $A^y_{m,n}$ | $A^z_{m,n}$ | $A^x_{m,n}$ | $A^y_{m,n}$ | $A^z_{m,n}$ |
| **(1,0)** | −0.0072 (3) | −0.0030 | 0.0011 | −0.00792 (8) | −0.00278 (11) | 0.00117 (3) |
| **(0,1)** | −0.0042 (2) | −0.0072 | −0.0011 | −0.00514 (14) | −0.00792 (8) | −0.00117 (3) |
| **(−1,1)** | 0.0030 | −0.0043 | 0.0011 (1) | −0.00278 (11) | 0.00514 (14) | −0.00117 (3) |
| **(1,1)** | 0.0012 (3) | −0.0014 | −0.0009 | 0.00150 (14) | −0.00161 (18) | −0.00103 (5) |
| **(−1,2)** | 0.0026 (3) | 0.0012 | 0.0009 | 0.0031 (2) | 0.00150 (14) | 0.00103 (5) |
| **(−2,1)** | 0.0014 | 0.0026 | −0.0009 (1) | 0.00161 (18) | 0.0031 (2) | −0.00103 (5) |
| **(2,0)** | −0.0014 (6) | −0.0001 | 0.0003 | −0.0015 (2) | −0.00018 (17) | 0.00028 (6) |
| **(0,2)** | −0.0012 (4) | −0.0014 | −0.0003 | −0.0013 (3) | −0.0015 (2) | −0.00028 (6) |
| **(−2,2)** | 0.0001 | −0.0012 | 0.0003 (1) | 0.00018 (17) | −0.0013 (3) | 0.00028 (6) |
| **(1,2)** | −0.0001 (9) | −0.0011 | 0.0005 | −0.0014 (3) | −0.0018 (2) | 0.00052 (8) |
| **(−2,3)** | 0.0010 (7) | −0.0001 | −0.0005 | 0.0004 (3) | −0.0014 (3) | −0.00052 (8) |
| **(−3,1)** | 0.0011 | 0.0010 | 0.0005 (2) | 0.0018 (2) | 0.0004 (3) | 0.00052 (8) |
| **(2,1)** | | | | 0.0012 (3) | 0.0011 (3) | −0.00001 (12) |
| **(−1,3)** | | | | 0.0001 (5) | 0.0012 (3) | 0.00001 (12) |
| **(−3,2)** | | | | −0.0011 (3) | 0.0001 (5) | −0.00001 (12) |

| | | | |
|---|---|---|---|
| **(3,0)** | −0.0001 (5) | 0.0006 (5) | −0.00002 (15) |
| **(0,3)** | −0.0007 (7) | −0.0001 (5) | 0.00002 (15) |
| **(−3,3)** | −0.0006 (5) | −0.0007 (7) | −0.00002 (15) |

**Table S10** Fourier series amplitudes of the cosine components ($B^x_{m,n}$, $B^y_{m,n}$, $B^z_{m,n}$) of the displacive modulation function for the S site (2/3, 1/3, 0.0862). Empty fields were not refined in the reference model.

| | **Spijkerman et al. (1997)** | | | **this study** | | |
|---|---|---|---|---|---|---|
| **(m, n)** | $B^x_{m,n}$ | $B^y_{m,n}$ | $B^z_{m,n}$ | $B^x_{m,n}$ | $B^y_{m,n}$ | $B^z_{m,n}$ |
| **(1,0)** | 0.0025 (3) | −0.0007 | 0.0029 | 0.00275 (8) | −0.00005 (11) | 0.00311 (3) |
| **(0,1)** | 0.0032 (2) | 0.0038 | 0.0029 | −0.00281 (13) | −0.00275 (8) | 0.00311 (3) |
| **(−1,1)** | 0.0007 | 0.0032 | 0.0029 (1) | 0.00005 (11) | 0.00281 (13) | 0.00311 (3) |
| **(1,1)** | −0.0032 (3) | −0.0012 | −0.0004 | −0.00354 (14) | −0.00117 (18) | −0.00039 (5) |
| **(−1,2)** | 0.0020 (3) | 0.0032 | −0.0004 | 0.0024 (2) | 0.00354 (14) | −0.00039 (5) |
| **(−2,1)** | 0.0012 | −0.0020 | −0.0004 (1) | 0.00117 (18) | −0.0024 (2) | −0.00039 (5) |
| **(2,0)** | 0.0010 (6) | 0.0031 | −0.0003 | 0.0004 (2) | 0.00327 (16) | −0.00039 (6) |
| **(0,2)** | 0.0021 (5) | −0.0010 | −0.0003 | 0.0029 (3) | −0.0004 (2) | −0.00039 (6) |
| **(−2,2)** | −0.0031 | −0.0021 | −0.0003 (1) | −0.00327 (16) | −0.0029 (3) | −0.00039 (6) |
| **(1,2)** | 0.0001 (9) | −0.0008 | −0.0003 | 0.0002 (3) | −0.0011 (2) | −0.00025 (8) |
| **(−2,3)** | −0.0010 (6) | −0.0001 | −0.0003 | −0.0013 (4) | −0.0002 (3) | −0.00025 (8) |
| **(−3,1)** | 0.0008 | 0.0010 | −0.0003 (2) | 0.0011 (2) | 0.0013 (4) | −0.00025 (8) |
| **(2,1)** | | | | 0.0003 (3) | −0.0003 (3) | 0.00020 (11) |
| **(−1,3)** | | | | −0.0006 (5) | −0.0003 (3) | 0.00020 (11) |

| | | | |
|---|---|---|---|
| **(−3,2)** | 0.0003 (3) | 0.0006 (5) | 0.00020 (11) |
| **(3,0)** | 0.0010 (5) | −0.0004 (4) | 0.00024 (15) |
| **(0,3)** | −0.0013 (7) | −0.0010 (5) | 0.00024 (15) |
| **(−3,3)** | 0.0004 (4) | 0.0013 (7) | 0.00024 (15) |

**Table S11** Dataset statistics and structure refinement parameters for the muscovite X-ray diffraction data recorded at PETRA III beamline P11.

| | |
|---|---|
| **Crystal / Sample** | |
| Chemical sum formula | $KAl_3Si_3O_{10}(OH)_2$ |
| *Z* | 2 |
| Crystal system | monoclinic |
| Space group | *C*2/*c* |
| Hall group | *-C 2yc* |
| *a*, *b*, *c* (Å) | 5.20, 9.03, 20.10 |
| *α*, *β*, *γ* (deg.) | 90, 95.8, 90 |
| *V* (Å³) | 939.4 |
| **Data collection** | |
| Diffractometer | Beamline P11, PETRA III, DESY, Germany |
| Radiation type | Synchrotron |
| Radiation wavelength (Å) | 0.61991 |
| Detector | Eiger 16M detector, Dectris (Switzerland) |
| Sample temperature (K) | 293 |
| Rotation range $\alpha_{min}$, $\alpha_{max}$ (deg.) | -50, 60 |
| Rotation range per saved frame (deg.) | 0.1 |
| $h_{min}$, $h_{max}$ | -7, 7 |
| $k_{min}$, $k_{max}$ | -12, 11 |
| $l_{min}$, $l_{max}$ | -17, 25 |
| Mosaicity (°) | 21.84 |
| **Data set statistics** | |
| High-resolution limit (Å) | 0.74 |
| Completeness (%) | 89.2 |
| Multiplicity | 2.4 |
| $<I/\sigma>$ | 41.2 |
| Measured reflections | 2362 |
| Unique reflections | 1039 |
| Unique reflections $I > 3\sigma$ | 1013 |
| **Refinement parameters** | |
| $R[F^2 > 2\sigma(F^2)]$, $wR(F^2)$, $S$ | 5.0%, 17.9%, 10.6% |
| No. of structural parameters | 105 |
| No. of refinement parameters | 92 |
| H-atom treatment | All H-atom parameters refined |
| $\Delta\rho_{max}$, $\Delta\rho_{min}$ (e Å$^{-3}$) | 1.516, −1.659 |

**Table S12** Deviation of atomic coordinates of the muscovite structures measured with MeV electron diffraction (this work), X-ray diffraction (this work), and Neutron diffraction by Gatta *et al.* (Gatta *et al.*, 2011*a*).

| Comparison (Bilbao Server) | avg. deviation of atomic positions of non-H (Å) | max. deviation of atomic positions of non-H (Å) | deviation of the atomic position of the H-atom (Å) |
|---|---|---|---|
| ED vs XRD | 0.0094 | 0.024 | 0.1567 |
| ED vs ND | 0.0320 | 0.083 | 0.0619 |
| XRD vs ND | 0.0263 | 0.061 | 0.1754 |

## S14. Additional supporting files

### S14.1. Movie S1.

Animated de Wolff section (xs1-xs4) of the Ta site from the ab initio structure solution. Contour lines shown in steps of 33 e/Å. The maximum electrostatic potential is about 260 e/Å. The line represents the model of atomic surface of Ta (brown) based on the kinematical refinement.

### S14.2. Movie S2.

Animated de Wolff section ($x_{s2}$-$x_{s4}$) of the Ta site from the ab initio structure solution. Contour lines shown in steps of 33 e/Å. The maximum electrostatic potential is about 260 e/Å. The line represents the model of atomic surface of Ta (brown) based on the kinematical refinement.

### S14.3. Movie S3.

Animated de Wolff section ($x_{s3}$-$x_{s4}$) of the Ta site from the ab initio structure solution. Contour lines shown in steps of 33 e/Å. The maximum electrostatic potential is about 260 e/Å. The line represents the model of atomic surface of Ta (brown) based on the kinematical refinement.

### S14.4. Movie S4.

Animated de Wolff section ($x_{s1}$-$x_{s4}$) of the S site from the ab initio structure solution. Contour lines shown in steps of 3.3 e/Å. The maximum electrostatic potential is about 25 e/Å. The line represents the model of atomic surface of S (yellow) based on the kinematical refinement.

### S14.5. Movie S5.

Animated de Wolff section ($x_{s2}$-$x_{s4}$) of the S site from the ab initio structure solution. Contour lines shown in steps of 3.3 e/Å. The maximum electrostatic potential is about 25 e/Å. The line represents the model of atomic surface of S (yellow) based on the kinematical refinement.

### S14.6. Movie S6.

Animated de Wolff section ($x_{s3}$-$x_{s4}$) of the S site from the ab initio structure solution. Contour lines shown in steps of 3.3 e/Å. The maximum electrostatic potential is about 25 e/Å. The line represents the model of atomic surface of S (yellow) based on the kinematical refinement.
